# Metabolic control analysis enables rational improvement of *E. coli* l-tryptophan producers but methylglyoxal formation limits glycerol-based production

**DOI:** 10.1186/s12934-022-01930-1

**Published:** 2022-10-04

**Authors:** Kristin Schoppel, Natalia Trachtmann, Emil J. Korzin, Angelina Tzanavari, Georg A. Sprenger, Dirk Weuster-Botz

**Affiliations:** 1 Institute of Biochemical Engineering, Technical University of Munich, Boltzmannstrasse 15, 85748 Garching, Germany; 2 Institute of Microbiology, University of Stuttgart, Allmandring 31, 70569 Stuttgart, Germany

**Keywords:** Metabolic control analysis, l-tryptophan, Methylglyoxal, *Escherichia coli*, Thermodynamics-based flux analysis, Glycerol

## Abstract

**Background:**

Although efficient l-tryptophan production using engineered *Escherichia coli* is established from glucose, the use of alternative carbon sources is still very limited. Through the application of glycerol as an alternate, a more sustainable substrate (by-product of biodiesel preparation), the well-studied intracellular glycolytic pathways are rerouted, resulting in the activity of different intracellular control sites and regulations, which are not fully understood in detail. Metabolic analysis was applied to well-known engineered *E. coli* cells with 10 genetic modifications. Cells were withdrawn from a fed-batch production process with glycerol as a carbon source, followed by metabolic control analysis (MCA). This resulted in the identification of several additional enzymes controlling the carbon flux to l-tryptophan.

**Results:**

These controlling enzyme activities were addressed stepwise by the targeted overexpression of 4 additional enzymes (*trpC*, *trpB*, *serB*, *aroB*). Their efficacy regarding l-tryptophan productivity was evaluated under consistent fed-batch cultivation conditions. Although process comparability was impeded by process variances related to a temporal, unpredictable break-off in l-tryptophan production, process improvements of up to 28% with respect to the l-tryptophan produced were observed using the new producer strains. The intracellular effects of these targeted genetic modifications were revealed by metabolic analysis in combination with MCA and expression analysis. Furthermore, it was discovered that the *E. coli* cells produced the highly toxic metabolite methylglyoxal (MGO) during the fed-batch process. A closer look at the MGO production and detoxification on the metabolome, fluxome, and transcriptome level of the engineered *E. coli* indicated that the highly toxic metabolite plays a critical role in the production of aromatic amino acids with glycerol as a carbon source.

**Conclusions:**

A detailed process analysis of a new l-tryptophan producer strain revealed that several of the 4 targeted genetic modifications of the *E. coli* l-tryptophan producer strain proved to be effective, and, for others, new engineering approaches could be derived from the results. As a starting point for further strain and process optimization, the up-regulation of MGO detoxifying enzymes and a lowering of the feeding rate during the last third of the cultivation seems reasonable.

**Supplementary Information:**

The online version contains supplementary material available at 10.1186/s12934-022-01930-1.

## Introduction

In order to achieve sustainability in the industrial synthesis of chemicals, a reorientation towards biobased approaches is both indispensable and promising. One field of application in which the benefits of metabolic engineering outweigh organic chemical synthesis is the biosynthesis of natural products having more complex structures, which are difficult to synthesize by chemical routes. Despite the significant progress in metabolic engineering over the last decades, a rational design of the microbial hosts is still challenging given their high complexity and regulations inside the cells, which cannot be analyzed and understood by in vitro analysis of single enzymes.

A detailed insight into the metabolic network and a profound understanding of intracellular processes substantially increase the probability of successful rational strain design. So-called “metabolic analyses” have been developed in order to collect large datasets in vivo during microbial production processes [1]. One option for conducting metabolic analyses is in the stationary mode, in which a perturbation of a reference state being studied is achieved by a change in substrate or substrate availability [2–4]. In the rapid media transition (RMT) method, cell broth is withdrawn at the reference state from the production process, usually operated as a fed-batch process, followed by a centrifugation step and the transfer of the separated cells into smaller stirred-tank bioreactors filled with fresh media [4]. During the minutes thereafter, the cells are exposed to different carbon sources, which are supplied by increasing feeding rates [2]. This causes a disturbance of the metabolism and, within a few minutes, new metabolic steady states can be established [1, 5]. Through rapid sampling [5], the cell reaction to the deflection can be captured, and the subsequent measurement of intra- and extracellular metabolite concentrations provides quantitative information about the dynamic changes [6, 7]. Disturbance of the production process can be avoided by the spatial separation of the short-term metabolic analyses from the process bioreactor [8]. Through parallelization of the analyses reactors, the amount of metabolic steady state data is multiplied [6]. Fluxome and metabolome data gained in these metabolic analyses can serve as underlying data for the calculation of metabolic control analysis (MCA). This mathematical method is used to identify rate-limiting steps within the considered metabolic pathways, and it enables the targeted rational modification of the producer strains [9]. The data processing for MCA includes the estimation of intracellular flux distributions and the determination of network-specific thermodynamic quantities. Significant advances in the field of flux analysis and thermodynamic network analysis have been achieved during recent years [10–13]. Recently, methods combining flux analysis with thermodynamic constraints have been developed [14, 15]. Thermodynamics-based flux analysis (TFA) can be used to eliminate flux distributions that are contradictory with respect to cell physiology as well as bioenergetics, and TFA allows the integration of quantitative metabolome data into the models [15].

For instance, l-tryptophan is a suitable candidate for microbial production by virtue of its relatively complex structure, which can be naturally synthesized by the model organism *Escherichia coli (E. coli)* and its wide, valuable application in the food, feed, and pharmaceutical industries [16–18]. However, l-tryptophan biosynthesis consumes significant cellular resources. Many enzymes are involved, several precursor metabolites are required, and the entire process is energetically expensive [19]. In order to minimize the metabolic burden, l-tryptophan biosynthesis is tightly regulated by different mechanisms like enzyme feedback inhibition, attenuation, and repression [16, 18–22]. These control sites must be overcome in order to produce l-tryptophan in profitable amounts. The rational strain design of *E. coli* has resulted in high maximum l-tryptophan yields of 0.227 g g_glucose_ which corresponds to almost 50% of the theoretical maximal yield on glucose [23]. Even though glucose is a relatively cheap substrate and easy to metabolize for the cells, its usage has several disadvantages. One major drawback is that glucose is mostly derived from agricultural products, so it competes with the food and feed industry for terrestrial land, which has socioeconomic implications [24–26]. To meet the highest demands in terms of sustainability and recycling, the utilization of alternative carbon sources should be actively pursued.

Glycerol is a renewable feedstock, which accrues from the rapidly growing biodiesel production sector as a waste stream [25]. Therefore, it is cheap, abundant, and able to enter *E. coli* cells by means of protein-assisted facilitated diffusion, and the metabolic pathways for its conversion already exist in *E. coli* [26–28]. Uptake by diffusion is beneficial since energetic molecules, which are needed for active uptake systems like the phosphotransferase system, are preserved and available for other metabolic pathways [29, 30]. The high reduction grade of glycerol in comparison to glucose is responsible for a higher theoretic l-tryptophan yield*,* which is also relevant to microbial production [24, 29, 31].

However, when alternative carbon sources like glycerol are used, the metabolic steady state differs from that of glucose utilization. Metabolic fluxes are routed differently, and reaction directions can even be reversed, resulting in the activity of various control sites and regulations as well as differing levels of metabolite pools. An important control site in glycolysis is the methylglyoxal pathway, which regulates intracellular phosphate availability and compensates for high levels of dihydroxyacetone-phosphate [32–34]. Starting from dihydroxyacetone-phosphate (DHAP), this pathway generates methylglyoxal (MGO) through the enzymatic activity of methylglyoxal synthase (mgsa), coded by the *mgsA* gene, and a phosphate residue is liberated at the same time [32]. MGO can be degraded by different glyoxalases and is further processed to d-lactate, from where it can re-enter central metabolism via pyruvate [35]. A variety of MGO detoxification mechanisms exist in *E. coli*, not all of which are understood in detail [34]. For example, adaptive laboratory evolution studies have been conducted in order to gain insights into these detoxification processes [36]. As an electrophile which damages proteins and DNA, MGO is extremely toxic for the cells and, when produced at high rates, it can also be released into the extracellular space [33, 36–39]. When the cells enter a state of imbalance, thus causing the accumulation of DHAP or phosphate deficiency, the activity of the MGO pathway is boosted [40]. Thus, for a short period, the cells are producing a toxic compound as an intermediate strategy for the survival of metabolic imbalance and to prevent cell death [40]. Abdallah, et al*.* proved the ability of *E. coli* inherent proteins to repair enzymes damaged by glycation through glyoxal and MGO [41].

In previous MCAs, the control mechanism inside the *E. coli* cells during l-tryptophan production was revealed, and the enzymes indole-glycerolphosphate synthase (igps), tryptophan synthase (trps2), 3-dehydroquinate synthase (dhqs), phosphoserine phosphatase (psp_L), and phosphoribosylpyrophosphate synthetase (prpps) were identified to limit l-tryptophan formation from glycerol in the *E. coli* strain NT1259 pF112*aroFBL*_Kan_, which was used in these analyses [42, 43]. Following up on these findings, the strains were genetically modified, and the production performance was validated in the standardized fed-batch production process. Thereupon, MCA with a modified producer strain was performed to analyze the shift of limitations within the cells. A special focus is placed upon the comparison of the metabolic states before and after genetic engineering.

## Results and discussion

### Process performances of modified *E. coli* strains

*E. coli* K-12 strain NT1259 (see Table 1, Materials and Methods), which has previously been analyzed [42, 43], served as a benchmark strain, and the design of all further genotypes was built upon this reference strain. This predecessor strain carries mutations in the structural gene *trpE*, which lead to a feedback-resistant version of anthranilate synthase (ANS), as well as one further mutation that is responsible for an overexpression of the genes in the *trp*-operon. The genes *trpL* (leader peptide), *trpR* (repressor gene), *sdaB* (l-serine deaminase), and *tnaA* (tryptophanase) were deleted. Furthermore, additional copies of the genes *tktA* (transketolase 1), *trpB-trpA* (tryptophan synthase), and a feedback-resistant enzyme variant of the gene *serA* (phosphoglycerate dehydrogenase) were integrated into the chromosome of NT1259.

**Table 1:** Strains, plasmid, and primers used in this work

Strain	Genotype	Source
NT1259	W3110 Δ*trp*L *trp*E ^FBR^ (M293T) N168D A478T C237R Δ*trp*R::FRT Δ*tna*A::FRT Δ*sda*B::FRT Δ*lac*:: P_tac_-*aro*F-B-L Δ*fuc*::P_tac_-*tkt*A Δ*xyl*::P_tac_-*ser*A^FBR^ (T372N) Δ*rib*::P_tac_-*trp*B-*trp*A	[42]
NT1259 *shiA*_*Cg*_	NT1259 *mal*::*shiA*_*Cg*_	[43]
NT1405	NT1259 ara::P_tac_-IGPs-*myc-t*	This work
NT1438	NT1259 *∆mal*::P_tac_-*trpB-A*	This work
NT1439	NT1405 *∆mal*::P_tac_-*trpB-A*	This work
NT1444	NT1439 *∆mtl*::P_tac_-*serB*	This work
NT1445	NT1439 *∆rec*A::P_t5_-*aroB*	This work
NT1446	NT1445 ∆*mtl*::P_tac_-*serB*	This work
pF112*aroFBL*_*Kan*_	*kan*^*R*^* colE lacI*^*q*^* P*_*tac*_*-aroFBL*	[7]
pCas	*RepA101*(Ts) *kan P*_*cas*_*-Cas9 P*_*araB*_*-Red lacI*^*q*^* P*_*trc*_*-*sgRNA*-pMB1*	[55]
pTarget-cat	*pMB1 cat*	[7]
pTarget-cat-_sg_Rib	*pMB1 cat sgRNA-Rib*	This work
pTarget-cat-_sg_Ara	*pMB1 cat sgRNA-Ara*	This work
pTarget-cat-_sg_Mal	*pMB1 cat sgRNA-Mal*	This work
pTarget-cat-_sg_Rec	*pMB1 cat sgRNA-Rec*	This work
pJF119-*trpBA*	*ColE1 amp;*	[7]
pJNNmod-*trpC*_*mt*_	*ColE1 amp; the synthesized fragment trpC (Mycobacterium tuberculosis) cloned into NdeI/HindIII sites under control of P*_*tac*_*-promoter*	This work
pJNNmod-*serB*	*ColE1 amp; the PCR fragment serB cloned into NdeI/BamHI sites under control of P*_*tac*_*-promoter*	This work
pJ-P_T5_-*aroB*	*ColE1 amp; the PCR fragment RBS-aroB was cloned into EcoRI/BamHI sites, P*_*tac*_* exchange to P*_*T5*_	This work

*Primers*	
sg-reverse	ACTAGTATTATACCTAGGACTGAGCTAGC
*rib*-integration1	ACCGTTCTTAATTCTGATATTTCATCGGTGATCTCCCGTCTGGGACATACCGATATCAAGGCGCACTCCCGTTCTGG
*rib*-integration2	ATTCACGCTAGCCCATACACCACGACTTCCTAAAGTAATCAGTACAGTACGGATACCCAGGGTTATTGTCTCATGAGCG
sgRib	GTATCGGTCAGCGCATCGGTGTTTTAGAGCTAGAAATAGCAAGTTAAAATAAGGCTAG
*ara*-integration1	TCTATAATCACGGCAGAAAAGTCCACATTGATTATTTGCACGGCGTCGTCGCTCAAGGCGCACTCCCGTTCTGG
*ara*-integration2	ATATAAGCGACCTCTTCCAGCACGATGGCGTTATGCACCGCATCTTCCGTCAGGATGGCCTTCTGCTTAATTTGATGCC
sgAra	GTCGCCAGCTTGATTGATCTGTTTTAGAGCTAGAAATAGCAAGTTAAAATAAGGCTAG
*mal*-integration1	AAGGTAAACTGGTAATCTGGATTAACGGCGATAAAGGCTATAACGGTCTCGCTGTCAAGGCGCACTCCCGTTCTGG
*mal*-integration2	GATCGGTAATGCAGACATCACGGCAGCGGCGGCAAAGTCACCCCACAGGTAGTTTTCAGGGTTATTGTCTCATGAGCG
sgMal	GTTGCCATCCGGCAGAATGGGTTTTAGAGCTAGAAATAGCAAGTTAAAATAAGGCTAG
*rec*A-integration1	TATAATTGCTTCAACAGAACATATTGACTATCCGGTATTACCCGTATCAAGGCGCACTCCCGTTCTGG
*rec*A-integration2	GACCCTTGTGTATCAAACAAGACGATTAAAAATCTTCGTTAGTTTCCCAGGGTTATTGTCTCATGAGCG
sgRec	CCCAGTTTACGTGCGTAGATGTTTTAGAGCTAGAAATAGCAAGTTAAAATAAGGCTAG
*mgsA*-RT-5'	GCATTTAGTGGATCCCAGAAGAAAATC
*mgsA*-RT-3'	AACGGCATCAACCGTTACTGGAAC
*gloA*-RT-5'	GCGTGGATAAATACGAACTCGGCAC
*gloA*-RT-3'	CAATTTTGTAACCGTCCGGATCTTCCAC
*gloB*-RT-5'	GTCCCTTCAAACAACCGACCACAC
*gloB*-RT-3'	GTCCACAAGAGACACAAGATAAGGGAAC
*gloC*-RT-5'	GCGGAACTGGCGCAACATTACG
*gloC*-RT-3'	CCTGTAAAGTCACATTCCCTATGCTG
*yeiG*-RT-5'	CATTACCGGATGTATGATTATCTGCGCG
*yeiG*-RT-3'	GCACGGATTCACAATTGGCGCAAAGG
*dld*-RT-5'	CGCGCGTATAAATGAAGACGGCAAAC
*dld*-RT-3'	CGGGCGTGTCGGCTTCAATATCAC
*yhbO*-RT-5'	AAAAGGAGAAGCCAGCGTGACCATCG
*yhbO*-RT-3'	TGATCAGCAACTGCGGGCCGTGAC
*yajL*-RT-5'	ACGATACGCCCGGAACGGTGGAACTG
*yajL*-RT-3'	CAGCGATGGTAACCTGGCGATTACC
*prs*-RT-5'	ACAACGCCGCCGATGTCCGGAG
*prs*-RT-3'	ACTGCGAAAGTGGTTGCAGACTTCC
*aroF*-RT-5ʹ	GCTGCAGATCGCGCGTAAATTGCTG
*aroF*-RT-3ʹ	CCGGAGGCCATTTCACGGTGAG
*trpC*-mt-RT-5'	TCTGGATAGCATTCTGGAAGGTGTTC
*trpC*-mt-RT-3'	GCTTGCACGTTTAACTTCGGCAATAAC
*trpC*-ec-RT-5ʹ	CTTATGACGCGGGCGCGATTTACGG
*trpC*-ec-RT-3ʹ	CCAGCGATAACACCTTAGCTTTGTCC
*serB*-RT-5'	AAGTAACCGAACGGGCGATGCG
*serB*-RT-3'	CCGGAGGCAATCGCCACTTTC
*aroB*-RT-5ʹ	GCATGGTCGCGATACTACGCTGG
*aroB*-RT-3ʹ	CGCGCCAATCATGTTTTTACCGAGG
*trpB*-RT-5ʹ	GTTTTACCCATCCGCTTCGCCAGCAAC
*trpB*-RT-3ʹ	AATTTCAGGCTCAGTTCAACGACCTGCTG

Based on the predecessor strain NT1259, new *E. coli* l-tryptophan producer strains were engineered according to the proposals made in [42, 43], which suggested increasing the expression of the genes *serB*, *prsA*, *trpC,* and *trpB*. Successful overexpression of the inserted genes was verified in shaking flask cultivations by means of RT-qPCR. Genetic modifications were introduced stepwise, and positive results were combined with further modifications. In addition to their chromosomal genotype, all strains carried plasmid pF112*aroFBL*_Kan_ [7] in order to improve flux through the shikimate pathway.

**Fig. 1:**
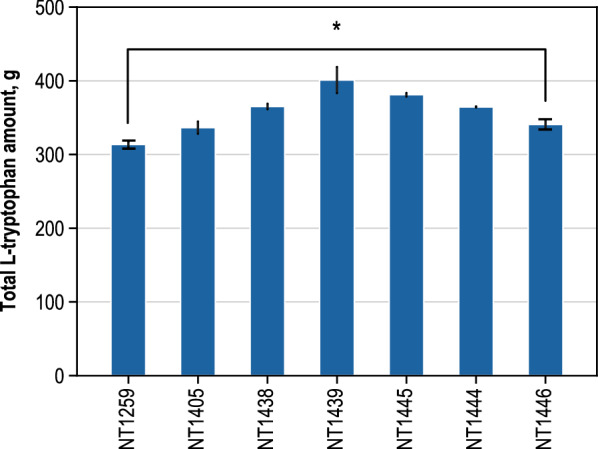
Final total l-tryptophan amounts (unit: g) in fed-batch cultivation processes on a 15 L scale (37 °C, pH 7.0, DO  > 30% air saturation) with *E. coli* strains (relevant genotype variations to the reference strain NT1259 are indicated: (NT1259), NT1259 *trpC*_*mt*_ (NT1405), NT1259 *trpBA* (NT1438), NT1259 *trpBA trpC*_*mt*_ (NT1439), NT1259 *trpBA trpC*_*mt*_* aroB* (NT1445), NT1259 *trpBA trpC*_*mt*_* serB* (NT1444), NT1259 *trpBA trpC*_*mt*_* aroB serB* (NT1446). All strains carried the plasmid pF112*aroFBL*_Kan_. Error bars without caps refer to standard deviations of different samples; error bars with caps represent the deviations of three technical replicates (*E. coli* NT1259 and *E. coli* NT1446). Level of significance*: 0.01 < p < 0.05

Cultivations of all *E. coli* strains were conducted in a standardized fed-batch production process on a 15 L scale, as proposed in [42, 43]. Sample handling was adjusted to also include precipitating l-tryptophan, which slightly influenced maximum product concentrations due to the washing off of adherent l-tryptophan from cells as compared to [42, 43]. Technical triplicates were realized for the reference strains *E. coli* NT1259 and *E. coli* NT1446 (additional chromosomal gene copies of *trpBA trpC*_*mt*_* aroB,* and *serB*). Single fed-batch cultivations were performed with all of the other strains. All of the strains were transformed with DNA of plasmid pF112*aroFBL*_Kan_ prior to cultivation. Figure 1 summarizes the fermentation process results of all the *E. coli* strains studied. The final l-tryptophan concentration was not able to be chosen for characterizing the process performances of the engineered *E. coli* strains because we observed a high level of stochastic process variability with respect to the end of l-tryptophan production in the fed-batch processes. An earlier end of the production phase led to lower final l-tryptophan concentrations due to the increasing dilution by means of ongoing constant substrate feeding and increased base additions for pH control, as compared to those processes having a stochastically prolonged production phase. The total final l-tryptophan amount produced in the bioreactor was easily accessible at the end of each fermentation process and was independent of the stochastically varying durations of the production phase. The l-tryptophan and CDW amounts produced throughout all of the fed-batch processes are provided in the Additional file 1: Figure S1–S7.

Using the reference strain *E. coli* NT1259, total final l-tryptophan amounts of 313.6 ± 4.9 g were achieved in the fed-batch process on a 15 L scale. Regarding this strain, two limiting steps were identified by MCA in the previous studies in the l-tryptophan specific biosynthesis route holding control over the formation of l-tryptophan [42, 43]. The enzymatic capacity of tryptophan synthase (trps2), which catalyzes the last step to produce l-tryptophan from l-serine and indole, was thereby estimated to be the strongest overall limitation on l-tryptophan biosynthesis, whereas only minimal control of l-tryptophan formation was estimated for Indole-3-glycerol-phosphate synthase (igps) [42, 43].

According to these findings in the first engineering steps separate *E. coli* strains were constructed with a second chromosomal copy of *trpBA* genes, coding for both subunits of tryptophan synthase (trps2 and trps3) (NT1438) and overexpression of *trpC* from *Mycobacterium tuberculosis* (*M. tuberculosis*)*,* which codes for a monofunctional igps (NT1405). No clear effect was observed when using *E. coli* N1405; the total l-tryptophan amount rose to a slightly higher value of 336.5 ± 10.1 g. However, when using *E. coli* NT1438 an improvement in process performance was achieved and, by combination of the genomic overexpression of *trpBA* and *trpC*_*mt*_ into *E. coli* NT1439, the total l-tryptophan amount further rose to 401.0 ± 21.7 g.

Next, dhqs (encoded by gene *aroB*) had been identified as a controlling step for l-tryptophan production by previously performed MCA [43]. Consequently, another copy of the corresponding *aroB* gene was introduced into the chromosome of *E. coli* NT1239, which resulted in *E. coli* NT1445. Another control site was estimated to occur in the l-serine supply. Specifically, psp_L was determined to be a critical step for l-tryptophan biosynthesis in the reference strain [42, 43]. To remove limitations in l-serine provision, the corresponding *serB* gene was inserted as a second gene copy in order to generate *E. coli* NT1444.

Using both strains (*E. coli* NT1445 and *E. coli* NT1444*)* only a negligible decrease in productivity compared to that of *E. coli* NT1259 was measured in the fed-batch process and resulted in total l-tryptophan amounts of 381.1 ± 3.0 g and 364.6 ± 0.9 g, respectively. The combination of overexpressed *aroB* and *serB* in *E. coli* NT1446*,* which then carried all 4 genetic modifications (*trpC*_*mt*_*, trpBA, aroB,* and *serB*), resulted in an average total l-tryptophan amount of 340.8 ± 7.8 g in the fed-batch. According to this result, not all of the genetic modifications which had been introduced as a follow-up of the former MCA, resulted in individual improvements in process performance. Most clearly, the insertion of *trpBA*-gene in combination with the *trpC*_*mt*_-gene affected productivity (Fig. 1). With *E. coli* NT1439, carrying those two modifications (*trpBA, trpC*_*mt*_), the final total l-tryptophan amount was 28% higher compared to the reference strain NT1259.

In spite of the process variations caused by the stochastic discontinuation of l-tryptophan production at process times between 65 and 76 h in the fed-batch processes, a slight but significant improvement of 9% in the final total l-tryptophan amount of *E. coli* NT1446, which was equipped with all of the proposed genetic modifications [42, 43], was observed, compared to the reference strain NT1259.

### Production of l-tryptophan from glycerol using ***E. coli*** NT1446 pF112aro***FBL***_Kan_

**Fig. 2:**
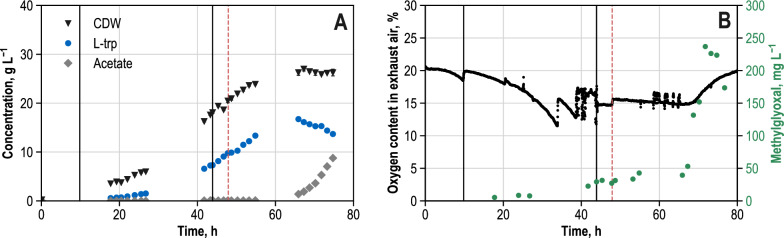
Fed-batch production of l-tryptophan with *E. coli* NT1446 pF112*aroFBL*_Kan_ on a 15 L scale (37 °C, pH 7.0, DO > 30% air saturation). **A**: Concentrations (unit: g L^−1^) of cell dry weight (CDW), L-tryptophan (l-trp) and acetate. **B**: Oxygen content in exhaust air (unit: %) and methylglyoxal concentration (unit: mg L.^−1^). Vertical solid black lines indicate (i) the end of the batch phase (9.83 h) and (ii) the beginning of the constant feeding phase/addition of IPTG (43.9 h). The dotted red line marks the process time for cell sampling for the parallel metabolic perturbation studies (47.9 h)

**Fig. 3:**
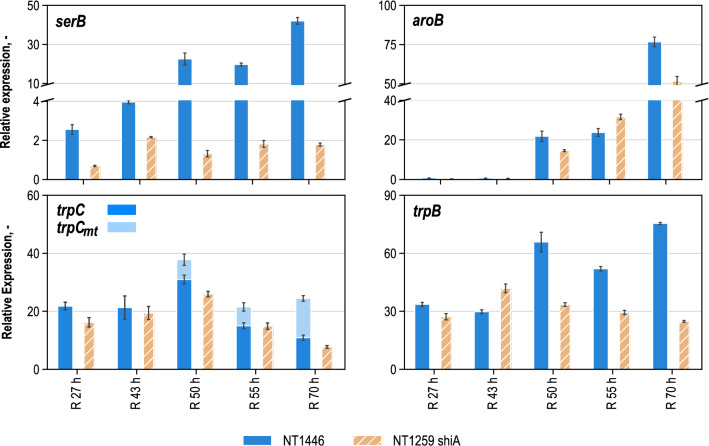
Gene expression (without units, relative to *ftsZ* control) of genes *serB*, *aroF*, *aroB*, *trpC*, *trpC*_*mt*_ and *trpB* relative to *ftsZ* gene of samples from reference L-tryptophan production process after 27 h (R 27 h), 43 h (R 43 h), 50 h (R 50 h), 55 h (R 55 h) and 70 h (R 70 h) process time (IPTG for the induction of inducible genes was added 44 h after inoculation. The samples were taken from the production process with NT1446 pF112*aroFBL*_Kan_ (NT1446) and likewise with NT1259 *shiA*_*Cg*_ pF112*aroFB*L_Kan_ (NT1259 shiA) [43]. Sample timepoints for NT1259 *shiA*_*Cg*_ pF112*aroFB*L_Kan_ were the same with maximum deviations of 1.5 h. Results for NT1259 *shiA*_*Cg*_ pF112*aroFB*L_Kan_ have been already published in [43]

*E. coli* NT1446 (equipped with plasmid pF112*aroFBL*_Kan_), which we will hereinafter abbreviate as *E. coli* NT1446, was chosen for further metabolic analysis. Among the strains available, the maximum possible change regarding the metabolic state was expected for this strain when compared to the predecessor strain *E. coli* NT1259 and to strain *E. coli* NT1259 *shiA,* which had been used in another prior study [43]. In this way, the effects of as many genetic modifications as possible on the fluxes relevant for l-tryptophan production were to be analyzed by means of a further MCA. The reference process on a 15 L scale used for metabolic analysis of strain NT1446 is shown in Fig. 2. RT-qPCR analyses were performed in order to be able to track expression of the genes which were edited in *E. coli* NT1446 (*serB, aroB, trpC and trpB*) during the course of the production process. Figure 3 depicts the results in comparison with the gene expression of strain *E. coli* NT1259 *shiA*. *E. coli* NT1259 *shiA*, carrying an additional *shiA* gene for the transport of shikimate, was used for direct comparison of the results in all subsequent matters because both the metabolic analyses and the metabolic control analyses were performed using improved methods and models, which had not been the case for *E. coli* NT1259. In addition, it had previously been shown that the expression of the transporter gene *shiA* did not influence process performance [43]. The fed-batch process remained unchanged compared to the *E. coli* NT1259 and *E. coli* NT1259 *shiA* strains [42, 43], but the sample handling was modified (see Material and Methods). During the initial batch and the subsequent exponential feeding phase, the cell dry weight (CDW) concentration rose to 18.10 ± 0.18 g L^−1^ with an average growth rate of µ = 0.07 h^−1^ (Fig. 2A). Growth diminished as of approximately 66 h of process time. Afterwards, CDW concentration only marginally increased to 26.26 ± 0.69 g L^−1^ until the end of the process. l-tryptophan production had already begun during the exponential feeding phase and 7.31 g L^−1^ l-tryptophan had already been formed at the end of this process phase (Fig. 2A). At this process time, feeding was switched to a constant rate of 0.2 g_gly_ g_CDW_ h^−1^ and 0.3 mM IPTG were added for the induction of P_tac_-controlled genes relevant to the l-tryptophan biosynthesis. l-tryptophan formation continued until a process time of 65.9 h to a maximum concentration of 16.73 g L^−1^. After that, l-tryptophan biosynthesis ended rather abruptly, and l-tryptophan concentration steadily declined to 12.29 g L^−1^ until the end of the process. 86% of this decrease in the product concentration can be explained by dilution through the continuous feeding and base addition. The reason for the remaining difference of 14% is unknown. It might be the consequence of chemical or physical l-tryptophan degradation [44], and catabolism of l-tryptophan should be unlikely due to the deletion of the gene coding for tryptophanase, *tnaA*. Considerable amounts of glycerol, which was provided as the sole carbon source, were detected in the fed-batch process after a process time of 70.2 h. Until the end of the process, glycerol accumulated to 7.22 g L^−1^ due to the continuous feeding and reduced uptake (Additional file 1: Figure S8). Ammonium concentration was kept above 2 g L^−1^ during the whole process (Additional file 1: Figure S8). At a process time of 65.9 h acetate started to accumulate; its formation continued until the end of the process with a final concentration of 8.76 g L^−1^. Only low maximum concentrations of l-tyrosine (0.12 g L^−1^) and l-phenylalanine (0.48 g L^−1^) were detected (Additional file 1: Figure S8). Throughout the process, concentrations of malate and pyruvate were below 1 g L^−1^. d-lactate concentrations rose to 2.2 g L^−1^ towards the end of the process (Additional file 1: Figure S8). The reasons for the formation of organic acids towards the end of the process, although the oxygen content in the exhaust air increased, remain unclear. High measured intracellular NADH/NAD^+^ ratios (> 0.1) at the timepoint of the metabolic analysis could be an indication for a disturbed electron transport chain.

Another byproduct that occurred during the cultivation of *E. coli* NT1446 was methylglyoxal (MGO; Fig. 2B), which is known to be highly toxic to cells [33, 39, 41]. Until a process time of 41.7 h, the MGO concentration remained below 10 mg L^−1^. Towards the end of the exponential feeding phase the MGO concentration inclined to 30 mg L^−1^. Until 65.9 h the concentration remained approximately constant, and after 67.2 h a rapid increase to a maximum concentration of 240 mg L^−1^ MGO was observed at a process time of 71.8 h. It is noteworthy that the oxygen content in the exhaust air (Fig. 2B) started rising rapidly some minutes after the rapid increase in MGO concentration, at a process time of 67.2 h. In parallel, the d-lactate concentration increased continuously.

Apparently, there is a temporal correlation between the decline in cellular respiration (increasing oxygen content in exhaust air) and the rise in MGO concentration. MGO concentrations of around 20 mg L^−1^ have been described in the literature as being inhibitory to growth. Above 40 mg L^−1^, cell death is induced accordingly [38, 39, 45]. During the cultivation of *E. coli* NT1446, MGO concentrations of above 20 mg L^−1^ were already measured towards the end of the exponential feeding phase, and extracellular MGO did not drop below the inhibitory threshold of 20 mg L^−1^ from 41.7 h until the end of the process.

It is probable that persistently high concentrations of MGO negatively affected the cell metabolism and may have caused continuously high stress levels. High intracellular DHAP concentrations as well as the ensuing production and detoxification of MGO may have caused a rising metabolic burden for the cells, which was further impeded by DNA and protein damage caused by MGO. Possibly, when stress exceeded a certain level, the metabolism shifted and the negative effect through MGO accumulation on the metabolism was irreversible, which may have led to the rapid collapse of l-tryptophan production and the reduction in cell respiration. From this process time on, the residual metabolic activity of the *E. coli* cells was put into minor growth and the formation of MGO and organic acid side-products. Glycerol assimilation to DHAP is regulated by enzymatic inhibition of glycerol kinase (glyk) by fructose 1,6-bisphosphate (FBP) [46]. Only when glyk becomes insensitive to FBP by protein engineering should the MGO production be released [37]. However, resequencing of the *glpK* gene from *E. coli* NT1446 showed no deviation from the wild type DNA sequence. The synthesis of MGO compensates for high levels of DHAP while simultaneously generating phosphate, which is why this pathway also regulates intracellular phosphate availability and is triggered when an intracellular phosphate limitation is prevalent [32, 34, 38, 47]. The extracellular phosphate levels never fell below 6.5 g L^−1^ during the process. However, no conclusion can be deduced with respect to intracellular phosphate availability, and therefore an intracellular phosphate limitation cannot be excluded as reason for the MGO formation.

Rising MGO concentrations were also measured in other cultivations with strains which were compared in the previous section. It might be reasonably assumed, that MGO formation led to the early and temporally unpredictable breakdown in production for all the L-tryptophan producing strains. Furthermore, the persistent stress levels caused by the toxicity of MGO during the production phase are crucial to the collapse of product formation.

The RT-qPCR results for the relative expression of the edited genes *serB, aroB, trpC* and *trpB* throughout the process are provided in Fig. 3. The expression of gene *serB* had quite clearly been boosted comparing the results of both strains. In *E. coli* NT1259 *shiA*, dhqs was identified as limiting product synthesis and, as a follow-up, a second copy of the corresponding gene *aroB* was integrated into the chromosome of *E. coli* NT1446. The increase in expression of *aroB* after induction was retarded in *E. coli* NT1446 compared to *E. coli* NT1259 *shiA*. But overall, apart from the sampling time point after 50 h, the expression of *aroB* was higher in *E. coli* NT1446 than in *E. coli* NT1259 *shiA*. Until the end of the process the expression of *aroB* further increased in both strains.

The additional gene copy of *trpC* used to eliminate the limitation in igps was realized by inserting a non-native copy of *trpC* from *M. tuberculosis (trpC*_*mt*_*)*, which codes for a monofunctional version of igps. Figure 3 illustrates the expression of the native *trpC* gene and the additional insertion *trpC*_*mt*_ in a stacked manner. Even though the combined transcript levels of *trpC*_*ec*_ and *trpC*_*mt*_ was increased in *E. coli* NT1446, the expression of *trpC*_*mt*_ itself was relatively low.

The enzymatic step catalyzed by trps2 had been calculated to limit L-tryptophan biosynthesis the most in *E. coli* NT1259, and NT1259 *shiA*, respectively [42, 43]. As a result, an additional gene copy of the corresponding gene *trpBA* was present in *E. coli* NT1446. As can be seen in Fig. 3, relative expression of *trpB* was doubled in the modified strain at most of the sampling timepoints. Especially during the constant feeding phase, when IPTG was added for induction, the expression for all the edited genes was successfully increased in *E. coli* NT1446 compared to NT1259 *shiA*.

### Metabolic analysis during l-tryptophan production

The metabolic state of *E. coli* NT1446 during l-tryptophan production was further investigated by means of parallelized short-term metabolic analysis. Cells were withdrawn from the reference process 4 h after induction and the start of constant feeding at a process time of 47.9 h (before the start of MGO increase; Fig. 2). After fast centrifugation and subsequent resuspension in fresh media, the cell suspension was distributed equally to four parallel 1 L stirred-tank bioreactors. As in [2, 42], glucose, glycerol, pyruvate, and succinate were used to induce a deflection within the cells. 12 metabolic steady states were established within 21 min through the application of a three-stage constant and limiting feeding profile in the analysis reactors. The 13th metabolic state was constituted by the reference process during the time of metabolic analysis. Intra- and extracellular metabolome was captured by rapid sampling, and the proteome is assumed to have been constant within the analysis time of 21 min [2, 4, 42].

#### Extracellular fluxome

**Fig. 4:**
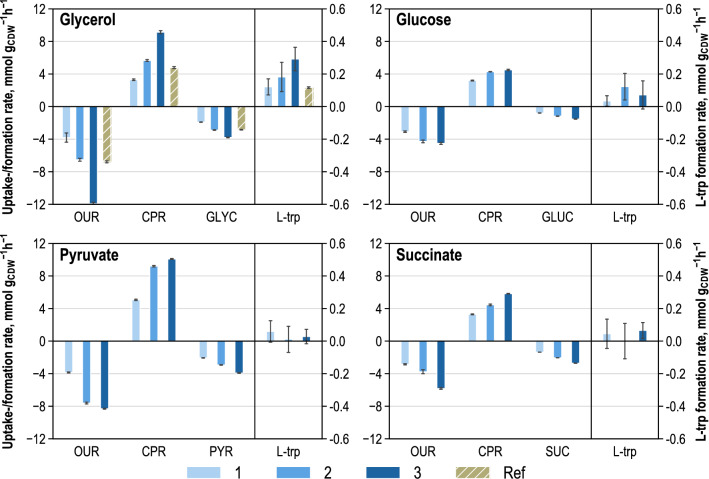
Extracellular uptake and formation rates (unit: mmol g_CDW_^−1^ h^−1^) of substrates, products, and respiration during parallelized short-term metabolic analysis of *E. coli* NT1446 pF112*aroFBL*_Kan_ producing l-tryptophan. Perturbation was achieved by three-stage feeding profiles containing each glycerol, glucose, pyruvate, and succinate. Respiration rates: oxygen uptake (OUR) and CO_2_ production (CPR); biomass specific substrate uptake rates of glycerol (GLYC), glucose (GLUC), pyruvate (PYR) and succinate (SUC) and biomass-specific production rates of l-tryptophan (l-trp) are depicted. Numbers 1–3: metabolic steady-state conditions, resulting from a corresponding three-stage feeding profile. Ref: Extracellular rates in the 15 L fed-batch reference process during metabolic analysis time

Sampling for the quantification of extra- and intracellular metabolites was conducted separately during metabolic analysis. Extracellular samples were simultaneously taken from the parallelized bioreactors at the beginning of metabolic analysis and at the end of each substrate supply phase. Additionally, sampling was performed from the reference process immediately before and after metabolic analysis. Extracellular biomass-specific rates for substrates, production, and by-product formation were calculated based on these data, and respiration rates were derived on the basis of online off-gas analysis. Figure 4 summarizes the extracellular rates for the uptake of substrates, production, and respiration. Biomass-specific rates from the reference process are included in the graph, which shows the results from the analysis reactor fed with glycerol.

Staggered respiration and substrate uptake rates were observed in all four analysis reactors, resulting from the three-stage feed profiles. The highest l-tryptophan formation rates were measured in the reactor having glycerol supply. The l-tryptophan production rates increased with an increasing feed supply to an overall maximum of 0.29 ± 0.08 mmol g_CDW_^−1^ h^−1^. This was not the case for the predecessor strain *E. coli* NT1259 pF112*aroFBL*_Kan_ (hereinafter abbreviated as NT1259) and for *E. coli* NT1259 *shiA* pF112*aroFBL*_Kan_ (hereinafter abbreviated as NT1259 *shiA*) [42, 43]. In the analysis reactor, which was supplied with glucose, the l-tryptophan formation increased from the first to the second feed stage and decreased again during the third. In the reactors fed with pyruvate and succinate, rather low l-tryptophan production rates were observed which were further significantly affected by errors.

Byproduct formation occurred especially during feeding stages 2 and 3. Ethanol formation was observed in all reactors operated at DO  > 50% air saturation, except for the analysis reactor, where pyruvate was used for perturbation. Ethanol was formed during the second and third feeding stage in the reactors having glucose and glycerol and during all feeding stages in the reactor having succinate feeding. Acetate, which was not washed out initially by centrifugation and resuspension, was taken up during the first feeding stage, due to high substrate limitation in all four analysis reactors. Later on, acetate was formed during the second and third feeding stage at rates of up to 0.5 mmol g_CDW_^−1^ h^−1^ in the reactors having glucose and pyruvate feeding. Increasing malate formation rates of up to 0.8 mmol g_CDW_^−1^ h^−1^ were measured in the reactor having succinate feeding. Constant malate formation rates of around 0.2 mmol g_CDW_^−1^ h^−1^ were observed in the analysis reactor having pyruvate supply. Formate was excreted in the analysis reactor supplied with glucose during the second feeding level at 0.67 ± 0.02 mmol g_CDW_^−1^ h^−1^ and the third at 0.55 ± 0.02 mmol g_CDW_^−1^ h^−1^. Succinate formation was not observed in any of the feeding stages of all four analysis reactors and in the reference process.

Furthermore, MGO formation was enzymatically quantified. With glucose used as the perturbation substrate, MGO rates of 0.046 ± 0.001 mmol g_CDW_^−1^ h^−1^ were determined during the second feeding level and of 0.018 ± 0.000 mmol g_CDW_^−1^ h^−1^ during the last. In the reactor with glycerol supply, MGO was formed at 0.002 ± 0.000 mmol g_CDW_^−1^ h^−1^ during the second supply level, and at 0.053 ± 0.001 mmol g_CDW_^−1^ h^−1^ during the third. In the two remaining analysis bioreactors (succinate and pyruvate) no MGO formation was observed given the gluconeogenetic growth. Therefore, high glucose and glycerol uptake rates appear to induce MGO biosynthesis. During glucose assimilation, the high initial production of MGO during the second feeding stage was reduced during the third. Conversely, the slight MGO formation rate with the perturbation substrate glycerol during the second feeding level was further increased by a factor of 26 during the last.

The substrate uptake, l-tryptophan production, and respiration rates for the reference process during metabolic analysis ranged between the lowest and intermediate rates of the analysis reactor having glycerol. The staggered uptake and production rates show that a deflection in both directions was successful.

#### Intracellular metabolome

In addition to extracellular fluxome, intracellular metabolite concentrations are necessary for the MCA. In addition, the intracellular metabolome provides a deep insight into the metabolic cellular processes. To enable quantitative metabolome analysis, special sampling with immediate cell inactivation was performed at the end of each substrate supply phase and concurrent with the four analysis bioreactors, and one additional sample was taken from the reference process on a 15 L scale.

**Fig. 5:**
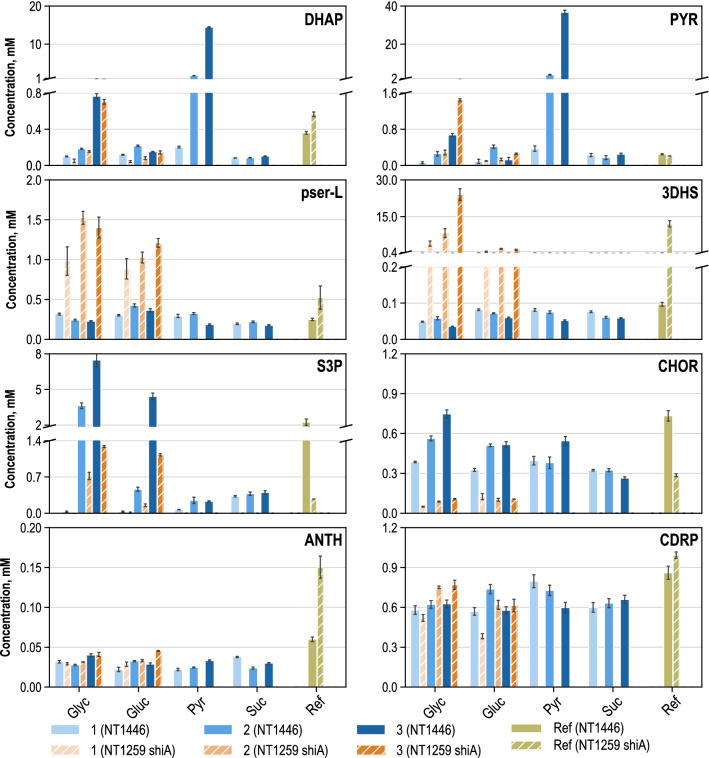
Intracellular concentrations (unit: mM) of dihydroxyacetone-phosphate (DHAP), pyruvate (PYR), o-Phospho-l-serine (pser_L), 3-dehydroshikimate (3DHS), shikimate 3-phosphate (S3P), chorismate (CHOR), anthranilate (ANTH), and 1-(2-carboxyphenylamino)-1-deoxy-d-ribulose 5-phosphate (CDRP) during parallelised short-term perturbation experiments in stirred-tank bioreactors with the carbon sources glycerol (GLYC), glucose (GLUC), pyruvate (PYR) and succinate (SUC). Numbers 1–3 (NT1446): steady-state conditions corresponding to three-stage feeding levels during metabolic analysis with *E. coli* NT1446 pF112*aroFBL*_Kan_. Ref (NT1446): intracellular concentrations in the reference fed-batch process with *E. coli* NT1446 during metabolic analysis. Numbers 1–3 (NT1259 shiA): steady-state conditions corresponding to three-stage feeding levels during metabolic analysis with the former strain *E. coli* NT1259 *shiA*_*Cg*_ pF112*aroFB*L_Kan_, published in [43]. Ref (NT1259 shiA): Intracellular concentrations in the reference fed-batch process with the former strain *E. coli* NT1259 *shiA*_*Cg*_ pF112*aroFB*L_Kan_ during metabolic analysis, published in [43]

In total, 45 metabolites were quantified which were crucial to l-tryptophan biosynthesis from glycolysis, glycerol metabolism, citric acid (TCA) cycle, pentose phosphate pathway (PPP), l-serine biosynthesis, and the synthesis of aromatic amino acids. To analyze and elaborate the metabolome changes in comparison to the preceding strain, an excerpt of the results from metabolic analysis with *E. coli* NT1446 is shown alongside the results for glycerol and glucose usage during metabolic analysis using *E. coli* NT1259 *shiA*. The NT1259 *shiA* results were already partially published in [43]. The intracellular metabolite concentrations of DHAP, pyruvate, O-Phospho-l-serine (pser_L), 3-dehydroshikimate (3DHS), shikimate 3-phosphate (S3P), chorismate (CHOR), anthranilate (ANTH), and 1-(2-carboxyphenylamino)-1-deoxy-D-ribulose 5-phosphate (CDRP) were selected for detailed discussion and are depicted in Fig. 5.

Increasing DHAP concentrations with rising substrate supply levels were measured in the analysis reactors fed with glycerol and pyruvate. No variations in the intracellular DHAP concentrations were observed in the cells fed with glucose and succinate. Comparing the results of *E. coli* NT1259 *shiA* and *E. coli* NT1446 during metabolic analysis, no significant differences were apparent with the substrates glucose and glycerol. The increasing DHAP concentrations were a direct consequence of glycerol assimilation and might have had an impact on the activation of the MGO pathway. DHAP accumulation also occurred when pyruvate was used for perturbation.

Intracellular pyruvate concentrations increased with a rising in glycerol supply, whereas the intracellular pyruvate concentrations in the analysis reactor with glucose feeding rose between the first and second feeding stage and decreased during the third. Intracellular pyruvate concentrations are also linked with the MGO synthesis pathway, since MGO can be degraded by different glyoxylases to d-lactate and can be further converted to pyruvate.

In previous MCA of *E. coli* NT1259 and *E. coli* NT1259 *shiA* [42, 43], psp_L was estimated to limit l-tryptophan production. In an attempt to eliminate the occurring bottleneck in psp_L, the corresponding gene *serB* was overexpressed in *E. coli* NT1446. Figure 5 shows that the pser_L level both during metabolic analysis and in the sample taken from the reference process with *E. coli* NT1446 was significantly decreased in comparison to NT1259 *shiA*. The genetic modification in l-serine biosynthesis appears to affect metabolite pools, thus indicating an enhanced conversion of pser_L to l-serine in NT1446.

By previous MCA of *E. coli* NT1259 and *E. coli* NT1259 *shiA*, respectively, dhqs had been identified to limit CHOR biosynthesis [42, 43] and, through enhanced perturbation, a slight control on l-tryptophan biosynthesis was revealed [43]. To remove this control site, the *aroB* gene was introduced as a second chromosomal copy present in strain NT1446. In comparing *E. coli* NT1259 *shiA* and *E. coli* NT1446, a clear shift in chorismate biosynthesis becomes apparent when examining the metabolites 3DHS, S3P, and CHOR. These changes were most likely induced by the overexpression of *aroB,* leading to an increased intracellular level of the downstream intermediates, S3P and chorismate.

Furthermore, additional gene copies of the genes *trpC*_*mt*_ and *trpBA* were present in *E. coli* NT1446. Metabolome quantification in this part of the tryptophan biosynthesis was rather difficult given the poor availability of purchasable standard solutions and the instability of the molecules. The CDRP levels were not influenced by the modifications inserted in *E. coli* NT1446, and the concentration remained nearly constant at around 0.6 mM during all metabolic steady states with both strains. It is possible that the CDRP producing reaction catalyzed by phosphoribosylanthranilate isomerase (prali) either still outpaced the reaction capacity of igps, or the effect of the extra gene copy of *trpC*_*mt*_ was marginal (see the section on transcript analysis).

### Metabolic control analysis

**Fig. 6:**
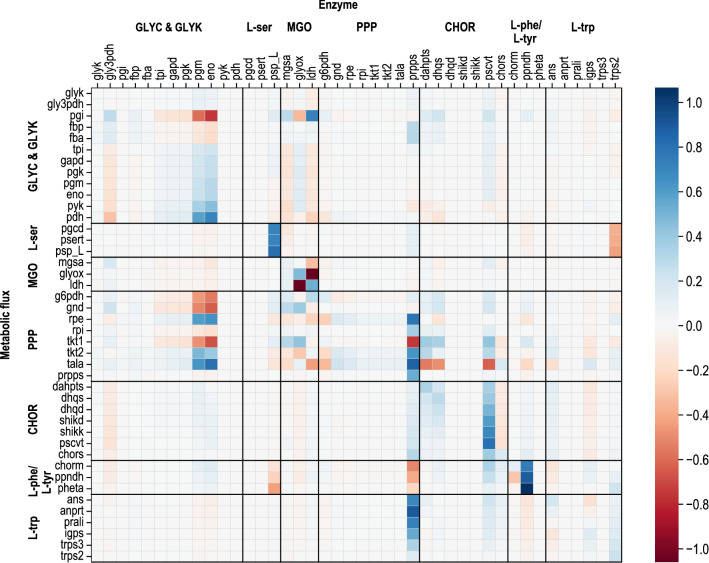
Mean flux control coefficients (unitless) estimated by metabolic control analysis of l-tryptophan production with *E. coli* NT1446 pF112*aroFBL*_Kan_. Columns represent enzyme activities, and lines refer to metabolic fluxes. The X-axis describes enzyme capacity; corresponding metabolic fluxes are represented on the Y-axis. The effects of changes in enzyme activity by one percent are illustrated (positive value: enhanced metabolic flux; negative value: reduced metabolic flux)

The metabolomics and fluxomics data for *E. coli* NT1446 provide deep insights into the cell metabolic state and indicate the position at which the metabolism was shifted through genetic modification. Nevertheless, due to the high complexity of the cellular network, no quantitative conclusions can be drawn based on only these results. Hence, MCA was applied to enable an accurate and quantitative description of control within the metabolic network considered, which was used to mathematically analyze the limitations inside the cell. Prior to MCA, we performed constraint-based modeling of intracellular flux distributions in consideration of the laws of thermodynamics, metabolite concentrations, and cell physiology, based on all of which intracellular metabolic fluxes and thermodynamic quantities were derived [15, 43]. The results for the intracellular flux distributions and Gibbs reaction energies are shown in Additional file 1: Figure S9–S10. MCA was then calculated according to the linearization approach proposed by Visser and Heijnen [48]. Flux control coefficients (FCCs), which were obtained from MCA, describe the effect whereby a steady-state metabolic flux responds to a change in a specific enzyme level. More precisely, the effect from a one percent increase in enzyme activity on the respective metabolic flux is represented by a FCC. Through normalization towards the reference state, FCCs become dimensionless, and values between −1.0 and 1.0 were obtained. Activating effects are described by positive FCC values, and inhibiting effects are represented by negative ones. Figure 6 summarizes FCCs for the network considered in the form of a heatmap, and the significant results are discussed in the following. A special focus will be on the FCCs relevant to l-tryptophan production.

A first control site appears in glycerol metabolism. Glycerol-3-phosphate dehydrogenase (gly3pdh) holds a minor positive control on reactions of gluconeogenesis and reactions from MGO pathway as well as the PPP and the opposite effect on chorismate- and by-product formation of l-phenylalanine and l-tyrosine. Negative control on CHOR biosynthesis pathway signifies that enhanced glycerol assimilation would not lead to an improved CHOR supply. The glycolytic enzymes phosphoglycerate mutase (pgm) and enolase (eno) showed strong controlling effects in a negative manner on gluconeogenesis and parts of the PPP and in a positive manner on glycolysis due to the competitive distribution of carbon flux in both directions.

In l-serine biosynthesis, psp_L was identified as having controlling effects on the metabolic network. It holds strong positive control towards all reactions in l-serine biosynthesis and negatively influences the by-product formation of l-phenylalanine and l-tyrosine. Negative control on l-phenylalanine and l-tyrosine synthesis can be explained by l-serine consumption for l-tryptophan biosynthesis. Put concisely, when l-serine production is improved by an up-regulation of psp_L, the adverse production of l-phenylalanine and l-tyrosine could be further reduced because the carbon flow would increasingly branch off into the direction of l-tryptophan. However, the positive control of psp_L on l-tryptophan production is very small, which is why the positive effect on productivity would be insignificant.

For MGO pathway enzymes, negative FCCs were estimated for mgsa and lactate dehydrogenase (ldh) towards reactions of glycolysis, whereas a positive control was estimated for the MGO detoxification enzyme glyoxylase (glyox). Controlling effects occurred within the MGO pathway. Furthermore, PPP was influenced moderately by the activity of MGO reactions, as the two pathways are also competing for the carbon flux originating from DHAP. However, when interpreting the results regarding the MGO pathway, the limitations of this method must be considered. Even though activating and inhibiting effects on single enzymes are included in the model, the global toxic impact of MGO on proteins and DNA in general is not comprised in the calculations. Therefore, the role of MGO biosynthesis for l-tryptophan production cannot be resolved by MCA alone.

Among the enzymes participating in PPP, only prpps has control over the metabolic network. As an extension of the classic PPP, prpps carries the enzymatic capacity to synthesize PRPP from R5P. PRPP serves as an important precursor metabolite in the L-tryptophan biosynthesis pathway and is therefore needed in high amounts. The far-reaching control of prpps on the whole metabolic network can be attributed to the key role of PRPP in l-tryptophan biosynthesis. Positive FCCs of prpps for l-tryptophan synthesis are very pronounced, thus indicating strong controlling effects towards product formation. Prpps has already been identified as being important control site by MCA of *E. coli* NT1259 and *E. coli* NT1259 *shiA* [42, 43]*.*

Several controlling effects were estimated in the CHOR biosynthesis. The first control was caused by 3-deoxy-7-phosphoheptulonate synthase (dahpts) reaching from PPP to CHOR biosynthesis; the same parts of the metabolism were slightly controlled by dhqs. 3-phosphoshikimate 1-carboxyvinyltransferase (pscvt) exerts strong control over the CHOR biosynthesis pathway and similarly limited control towards l-phenylalanine, l-tyrosine, and l-tryptophan biosynthesis. Regarding chorismate synthase (chors), a slight negative control on CHOR synthesis and marginal positive controlling effects were estimated towards l-tryptophan biosynthesis. In the by-product synthesis pathway, prephenate dehydratase (ppndh) had strong positive control towards all metabolic fluxes in this pathway branch and a slight negative control towards l-tryptophan biosynthesis. Downregulation of ppndh could contribute to shifting the metabolic flux further in the direction of the l-tryptophan branch by virtue of the competitive usage of CHOR. In l-tryptophan biosynthesis, a minor positive control of anthranilate synthase (ans) was observed on CHOR biosynthesis and ans itself as well as on anthranilate phosphoribosyltransferase (anprt). Negative controlling effects were estimated downwards from igps. This reverse effect was probably caused by the feed-forward inhibition of igps by ANTH [49]. The opposite effects occurred for igps and can also be associated with the influence of ANTH on igps [50]. Furthermore, the controlling influence of the last enzyme in l-tryptophan biosynthesis, trps2, emerges in particular in the l-tryptophan biosynthesis pathway, where it exercises weak positive control.

**Fig. 7:**
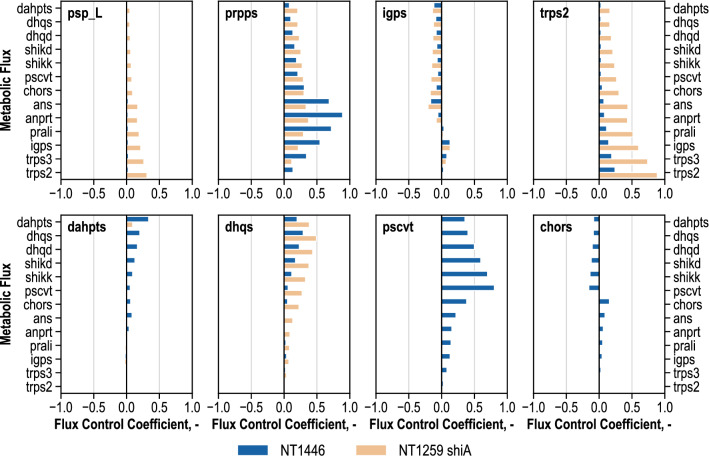
Selection of mean flux control coefficients (unitless) estimated by metabolic control analysis of *E. coli* NT1446 pF112*aroFBL*_Kan_ (NT1446) and NT1259 *shiA*_*Cg*_ pF112*aroFB*L_Kan_ (NT1259 shiA) during l-tryptophan production depicted for the enzymes phosphoserine phosphatase (psp_L), phosphoribosylpyrophosphate synthetase (prpps), indole-glycerolphosphate synthase (igps), tryptophan synthase (trps2), 3-deoxy-arabino-7-phosphoheptulosonate synthase (dahpts), 3-dehydroquinate synthase (dhqs), 3-phosphoshikimate 1-carboxyvinyltransferase (pscvt) and chorismate synthase (chors). Metabolic fluxes of chorismate and l-tryptophan biosynthesis are represented on the Y-axes. The effects of changes in enzyme activity by one percent are illustrated (positive value: enhanced metabolic flux; negative value: reduced metabolic flux). Results for NT1259 *shiA*_*Cg*_ pF112*aroFB*L_Kan_ were previously published in [43]

For the discussion of these results, the focus will be on the comparison of metabolic control during l-tryptophan production before and after genetic modification. Figure 7 summarizes the notable results regarding the metabolic fluxes of CHOR and L-tryptophan biosynthesis.

In *E. coli* NT1259 *shiA*, and NT1259, respectively, a limitation in psp_L towards l-tryptophan production had occurred [42, 43] which was addressed by the genomic insertion of an additional gene copy of the corresponding gene *serB* in *E. coli* NT1446. As can be seen in Fig. 7, the former limitation in psp_L is eliminated in strain NT1446, which was most likely due to the further overexpression of *serB*. This result coincides well with the increased *serB* expression level and the decreased intracellular levels of pser-L in *E. coli* NT1446.

In MCAs with the earlier strains, prpps was identified as limiting l-tryptophan production [42, 43]. Thereupon, the genomic overexpression of the corresponding *prsA* gene was suggested, but integration into NT1446 has for unknown reasons not been successful thus far. Hence, the metabolic control for this enzyme is not reduced in comparison with both strains. On the contrary, the control in prpps on metabolic fluxes of l-tryptophan formation was actually increased. The comparison of the expression analysis of both strains during the course of the production process shows that the expression of *prsA* was in fact decreased in *E. coli* NT1446, which might be the explanation for the increase in the controlling effect of the enzyme prpps on l-tryptophan production (data not shown).

Within the l-tryptophan biosynthesis pathway, igps was identified to limit production. An extra copy of *trpC* from *M. tuberculosis* was present in *E. coli* NT1446. However, MCA revealed that the genetic modifications of the producer strain *E. coli* NT1446—including the additional gene copy of *trpC*_*mt*_—did not resolve the control over l-tryptophan biosynthesis. This might be due to a persistent forward inhibition of igps by ANTH, which was discovered recently [49]. The expressed *trpC*_*mt*_ either did not show enough activity, did not circumvent forward-inhibition by ANTH, or perhaps the expression level of the non-native gene was insufficient for carrying an increased flux.

Moreover, the last enzyme in l-tryptophan biosynthesis (tryptophan synthase) had been calculated to hold the strongest control over l-tryptophan formation in *E. coli* NT1259 and NT1259 *shiA* [42, 43]. Through second copies of *trpBA* genes, the FCCs were reduced by around 75% in *E. coli* NT1446. Accordingly, the limitation in trps2 was certainly lowered, but not entirely eliminated. In the former strain NT1259 *shiA*, dhqs was calculated to positively control chorismate synthesis, and the controlling effect of dhqs reached into l-tryptophan biosynthesis. To tackle the control site in dhqs, a second copy of *aroB* was also present in *E. coli* NT1446. As a consequence of the genetic modifications present in *E. coli* NT1446, several changes occurred in the distribution of metabolic control within the CHOR biosynthesis pathway. Amongst other alterations, the metabolic control of dhqs on chorismate and l-tryptophan biosynthesis was reduced in *E. coli* NT1446. Furthermore, a marginal new limitation of dahpts towards CHOR synthesis was estimated, and a new control site appeared in the enzymatic capacity of pscvt. This enzyme positively controls CHOR synthesis with FCCs up to 0.8, and the limiting effects reach down to trps3. Moreover, a new small level of control was estimated for chors, and towards CHOR biosynthesis it appeared in a negative manner, whereas fluxes in l-tryptophan biosynthesis were affected positively. The estimated metabolic shift, which was induced by genetic modification of the producer strain, is emphasized by the differing metabolite concentrations within the chorismate biosynthesis pathway in comparison with both strains.

### Relative expression of targeted genes connected to the methylglyoxal pathway

**Fig. 8:**
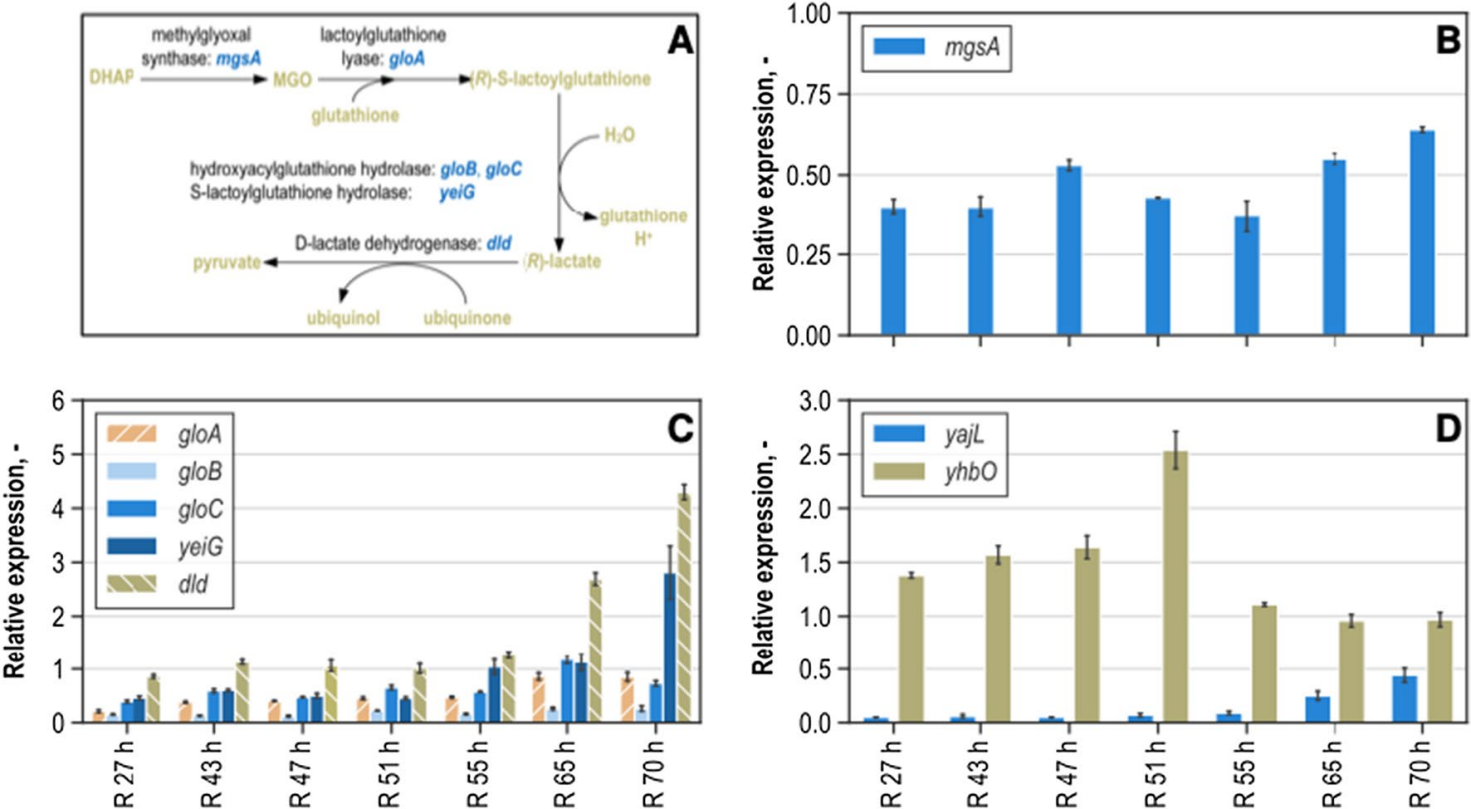
Schematic representation of the main methylglyoxal formation and degradation pathway in *Escherichia coli* [51] **A** and RT-qPCR analysis of relative gene expression (without unit) of genes *mgsA* (**B**), *gloA, gloB, gloC, yeiG, dld* (**C**), *yajL,* and *yhbO* (**D**) relative to *ftsZ* gene of samples from the reference l-tryptophan production process with NT1446 pF112*aroFBL*_Kan_ after process times of 27 h (R 27 h), 43 h (R 43 h), 50 h (R 50 h), 55 h (R 55 h), and 70 h (R 70 h). IPTG for the induction of inducible genes was added 44 h after inoculation

Close attention was also paid to the role of MGO in the l-tryptophan production process. The respective expression levels of genes coding for the enzymes involved in MGO pathway were analyzed in order to gain insight into the underlying processes of MGO formation and detoxification. The relative expressions of the corresponding genes *mgsA, gloA, gloB, gloC, yeiG, dld, yajL,* and *yhbO* are depicted in Fig. 8.

MGO synthesis is mainly carried out by mgsa [32, 39]. Relative expression of the corresponding gene *mgsA* (Fig. 8B) is rather stable throughout the l-tryptophan production process, but increases were measured at a process time of 47 h and towards the end of the process (65 h and 70 h). The rise in expression correlates well with the measured extracellular MGO concentrations, in which increased levels after the time of induction and very high concentrations after a process time of 70 h were measured. The main pathway for the detoxification of MGO used by *E. coli* is depicted in Fig. 8B and comprises the enzymes coded by the genes *gloA, gloB, gloC, yeiG,* and *dld.* Expression levels for all the genes participating in the detoxification of MGO were stable throughout the process. Only towards the end did the expression levels rise. A clear rise in expression was measured for all the genes participating in the detoxification of MGO, except for *gloB*.

Furthermore, the expression of the genes *yajL* and *yhbO* were analyzed, both of which belong to the repair enzymes capable of reversing damage from glycation induced by MGO [41]. Expression of *yajL* was relatively low compared to *yhbO* throughout the whole process. A slight increase in expression of *yajL* was measured at the very end of cultivation. Interestingly, the expression of *yhbO* exponentially increased until a process time of 51 h. In the following hours, until 55 h, the expression fell to a level below the expression during the first third of the fed-batch cultivation, although the MGO concentration rose drastically during the last third of the cultivation.

Expression analysis results for the analysis reactors are shown in the Additional file 1: Figure S11–S13. The remarkable results are discussed in the following. Expression of *mgsA* was 65% higher in the sample taken at the end of metabolic analysis from the reactor with glucose feeding in comparison with the analysis reactor having glycerol supply. The expression measured for the detoxification gene *gloA* was slightly reduced in the sample taken from the glucose analysis reactor in comparison with the one having glycerol supply. However, the expression of the remaining genes involved in the MGO degradation pathway was significantly higher when glucose was used as a perturbation substrate as compared to glycerol usage: 89% for *gloB*, 75% for *gloC*, 94% for *yeiG* and 30% for *dld*. A remarkably high expression of 2.41 for the gene *yhbO,* which codes for a glycation repair enzyme, was measured in the sample taken from the analysis reactor fed with glucose as compared to the sample from the analysis reactor having glycerol supply (0.73). The expression of *yhbO* differentiated significantly, by 230%. The expression of *yajL* was only increased by 20% in comparison with the glucose to glycerol supply.

Reduced expression of most MGO detoxifying and repair enzymes during glycerol assimilation compared to glucose usage could be a possible explanation for the significant drop in MGO production during glucose assimilation at increased substrate supply rates, as well as the further increase of extracellular MGO concentrations when glycerol was supplied at higher rates. Furthermore, these observations concur with the results by Tötemeyer et al. [47] and support the hypothesis that the MGO pathway is controlled differently during glucose and glycerol assimilation.

## Conclusions

Detailed insights by metabolome, expression, and computational fluxome analysis combined with MCA revealed deep insights into intracellular processes and enabled an improved comprehension of l-tryptophan production with *E. coli* when using glycerol as a sole carbon source. By virtue of rational strain design, several previously identified limitations [42, 43] were addressed, and their efficacy was evaluated. Using the new producer strain *E. coli* NT1446, which carried 4 of the supposed genetic modifications (*trpC*_*mt*_*, trpBA, aroB, serB*), total final l-tryptophan amounts of 340.8 ± 7.8 g on a 15 L scale and l-tryptophan yields on C-mol basis after 72 h of 14.33 ± 0.56% (mol C_L-trp_ (mol C_Gly_)) were achieved in the fed-batch process. This corresponds to an improvement of 9% in final total l-tryptophan amount and 6% in the carbon yield in comparison with the reference strain *E. coli* NT1259. Particularly by means of the detailed metabolic analysis in combination with MCA and expression analysis, the specific intracellular effects of the genetic modifications were disclosed, and a better understanding of the underlying processes was enabled.

The insertion of an additional copy of *prsA* gene, which was intended to tackle the limitations in prpps, has not yet been successful, so it was not applied in the improved producer strain. Further research is needed in order to optimize *prsA* integration and expression, and to dissolve the remaining strong control of prpps on l-tryptophan biosynthesis.

The limitation of psp_L, which is encoded by the *serB* gene, was fully resolved according to the MCA results. This finding was supported by the metabolome and expression analysis results. MCA revealed that metabolic control within CHOR synthesis was shifted away from dhqs, probably by overexpression of the *aroB* gene, whereby other moderate limitations appeared. Those could be addressed by further overexpressions of, e.g., the *aroA* gene.

Control of igps towards CHOR and l-tryptophan biosynthesis was not influenced by the genetic modifications, including the genomic integration of a *trpC* copy from *M. tuberculosis*. It is possible that the forward inhibition of ANTH towards the igps was not circumvented by the gene variant used. In a future attempt, a forward-activated igps from *Aspergillus niger* should be introduced into the producer strain, as was proposed by [49].

The limitation of trps2 on l-tryptophan formation was released in *E. coli* NT1446 by newly introduced genetic modifications, most likely by the insertion of an additional *trpBA* gene copy. The MCA and expression analysis results suggest that a further increase of trps2 activity by the further overexpression of *trpB* or alternative, strong promotors could boost l-tryptophan production.

A closer look at MGO production and expression analysis of the genes involved in the MGO pathway indicates that the metabolism of *E. coli* lost its balance several hours after induction due to unknown reasons and was shifted towards MGO production. At some point, MGO production was prevalent, and the expression of the required genes for the detoxification process then lagged. As a result, the cells metabolic counteractions ran too long, which led to a rapid termination of l-tryptophan production as well as reduced cell respiration. Comparing the expression analysis results from the respective degradation and repair enzymes provided hints that the detoxification of MGO is regulated differently than glucose and glycerol assimilation. In publications by Tötemeyer, et al. [47] *E. coli* mutations overexpressing the *mgsA*-gene exhibited normal growth during glucose conversion and no substantially increased MGO production; growth on glycerol was severely impeded and MGO accumulated in the medium. These findings suggest a very tight metabolic control of MGO biosynthesis during glucose usage in comparison with glycerol, thus indicating glycerol assimilation as an influencing factor in the activation of this pathway [40]. Although MGO formation had not until now been observed in fed-batch processes using glycerol as a carbon source [2, 7, 52, 53], no clear evidence of its absence has been provided. The high reactivity of MGO, its low molecular mass, and the lack of ultraviolet chromophore all hamper the detectability of low concentrations by means of conventional analytical detection systems like UV–VIS spectroscopy or refractive index detectors [54]. As a result, the role of MGO in microbial bioprocesses using glycerol as a carbon source has thus far remained unclear.

It is plausible that the sudden end of production was related to MGO formation in this fed-batch process—and possibly also in all the other processes using l-tryptophan producers conducted both in this present work and in previous studies [7, 42, 43]. The persistently moderate levels of MGO during the production phase probably have a major influence on the stress level and could be an explanation for the differences in production times, and thus on high variability of overall productivity. Furthermore, it seems likely that the l-phenylalanine production processes on glycerol as sole carbon source [2] are also affected by MGO production.

As a result, the upregulation of MGO detoxifying genes should be considered in the production of aromatic compounds as a possible measure for avoiding an early production break-off when glycerol is being used as the sole carbon source. Furthermore, from a bioprocess engineering perspective, it appears reasonable to lower the glycerol supply rates during the last third of the production process in order to avoid high intracellular DHAP concentrations, thus suppressing the MGO synthesis as long as possible.

## Materials and methods

### Strains, plasmids, and primers

The strains used in this work were developed using the CRISPR-Cas method, as described earlier by Jiang, et al. [55]. The plasmids pTarget-cat-sgRib, pTarget-cat-sgAra, pTarget-cat-sgMal were constructed by inverse PCR with the specific “sg-RNA -” and “sg-reverse” primers (see Table 1). The fragment containing P_tac_-*trpB-trpA*, P_tac_-*trpC*_*mt*_, P_tac_-*serB*, P_T5_-*aroB*, flanked with homology regions (about 40 nt), were amplified from pJF119-*trpBA*, pJNNmod-*trpC*_*mt*_, pJNNmod-*serB* and pJ-Pt5-*aroB* plasmids (sequences are presented in Suppl. materials) respectively with suitable primers (Table 1). The PCR fragments were introduced directly into the chromosome of the *E. coli* NT1259 strain by means of homology recombineering using the λ-Red system, cloned on the pCas plasmid. The selection of integrants was carried out on the MacConkey plates containing the corresponding sugar (1%), as well as kanamycin (50 µg/ml) and chloramphenicol (25 µg/ml). The integration accuracy was verified by means of PCR and sequencing of the integrated fragments.

### Growth medium

A minimal medium adapted from Albermann et al. [56] was used for the cultivation of *E. coli* strains. Heat-resistant components (g L^−1^): 3.00 KH_2_PO_4_, 12.00 K_2_HPO_4_, 5.00 (NH_4_)_2_SO_4_, 0.10 NaCl were dissolved in water and, after autoclaving, heat-labile ingredients (g L^−1^): 0.30 MgSO_4_·H_2_O, 0.015CaCl_2_·2H_2_O, 0.1125 FeSO_4_·7H_2_O, 1.50 sodium citrate, 0.0075 thiamine, and 0.05 kanamycin were added aseptically after cooling. Glycerol was autoclaved separately in a stock solution of 1000 g L^−1^, and 7 g L^−1^ were added to the medium for precultures and 4 g L^−1^ to the medium for cultivation on a 15 L scale. Additionally, the process medium was supplemented with 1 mL L^−1^ of a trace-element solution [57], containing (g L^−1^) 11.2 MnSO_4_·H_2_O, 10.0 AlCl_3_·6H_2_O, 7.3 CoCl_2_·6 H_2_O, 2.0 ZnSO_4_·7 H_2_O, 2.0 Na_2_MoO_4_·2 H_2_O, 1.0 CuCl_2_·2 H_2_O, 0.5 H_3_BO_3_.

### Fed-batch production process on a 15 L scale

The identical process as described by Tröndle et al. [42, 43], was used for the production of l-tryptophan on a 15 L scale. For the preparation of starter cultures, single colonies were picked from a lysogeny broth agar plate to inoculate two 100 mL shake flasks with 10 mL minimal medium each. Shake flasks were incubated for 40 h at 37°, shaking at 100 min^−1^ in an orbital shaker (Multitron incubation shaker, Infors HT, Bottmingen, Switzerland). The cell suspension was then distributed equally to ten 500 mL shake flasks, each of them containing 100 mL minimal medium. After another 20 h of incubation, orbitally shaking at 37 °C and 250 min^−1^, cell suspension was transferred into a sterile bottle. Before inoculation, the bioreactor was filled with basal medium, and sterilized in-situ (120 °C, 20 min). After cooling to 37 °C, the remaining heat-labile medium components were added aseptically. For the inoculation of the production process, the cell suspension was pumped aseptically into the 42 L stainless-steel stirred-tank bioreactor (Techfors, Infors HT, Bottmingen Switzerland), equipped with four equidistant baffles and three six-bladed Rushton impellers. For inoculation, 1.0 L of the preculture cell suspension was pumped into the stirred-tank bioreactor. The initial volume was adjusted to 15.0 L. During the whole process, the pH was maintained at 7.0 by titration of 25% NH_4_OH and 42% H_3_PO_4_, and the dissolved oxygen concentration was kept above 30% air saturation by adapting the stirrer speed (200–1000 min^−1^), aeration rate (5–40 L min^−1^), and pressure (up to 1.3 bar). An exhaust gas analyzer (Easy Line, ABB Automation, Zurich Switzerland) was used to monitor oxygen uptake and carbon dioxide formation.

The initial batch phase was followed by two consecutive exponential feeding phases: 1.0 L feeding solution A with (g L^−1^) 120.0 glycerol, 60.0 (NH_4_)_2_SO_4_, and 0.1 kanamycin, and thereafter 4.5 L feeding solution B with (g L^−1^) 400.0 glycerol, 25.0 (NH_4_)_2_SO_4_, and 0.1 kanamycin. Subsequently, a volumetrically constant feeding phase of feed solution C (5.0 L) containing (g L^−1^): 800.0 glycerol, 8.0 (NH_4_)_2_SO_4_, 8.0 (NH_4_)_2_HPO_4_, and 0.1 kanamycin, was applied. The feed rate was adjusted to the biomass concentration measured at the end of the exponential feeding phase to a value of 0.2 g_glycerol_ g_CDW_^−1^. At the beginning of the constant feeding phase 0.3 mM Isopropyl-β-D-thiogalactopyranosid (IPTG), as well as the initial amounts of MgSO_4_·H_2_O, CaCl_2_·2 H_2_O, FeSO_4_·7 H_2_O, and thiamine, were injected into the bioreactor aseptically.

### Parallelized perturbation experiment for metabolic analysis

The 15 L fed-batch cultivation with *E. coli* NT1446 served as a reference process for steady-state parallelized metabolic analysis. During the constant feeding phase, 3.6 L of cell suspension was withdrawn 4 h after induction through the bottom valve of the 42 L stirred-tank reactor. According to the rapid media transition method [4], cells were rapidly centrifuged (3260 g, 7.5 min, 37 °C; Rotixa centrifuge 50 RS, Hettich Zentrifugen, Tuttlingen, Germany), the supernatant was discarded, and cells were resuspended in 400 mL fresh minimal medium (37 °C) without carbon source.

The cells were then distributed in equal volumes to four parallel stirred-tank bioreactors (DASGIP technology/ Eppendorf, Jülich, Germany), which had been prefilled with 400 mL of fresh minimal medium, 0.3 mM IPTG, and 0.1% antifoam. During the 21 min of analysis time, the pH and dissolved oxygen concentration were measured using both individual probes and separate pumps for pH control, feed supply, gas mixing for air, N_2_, and O_2_, and separate gas analyzers. The pH was adjusted to 7.0 by the addition of 21% H_3_PO_4_ and 2 M NaOH. Temperature, gassing, and stirrer speed were kept constant at 37 °C, 4 L min^−1^ (50% O_2_ (v/v)), and 1200 min^−1^ respectively, throughout the metabolic analysis.

To achieve different metabolic steady-states, various substrates were supplied by a three-stage constant feeding profile. The feed supply rates of all reactors were increased 9.0 and 15.0 min after inoculation. The first reactor was provided with a 62 g L^−1^ glucose feeding solution, and the supply rates were set to 31.7 mL h^−1^, 63.4 mL h^−1^, and 95.1 mL h^−1^. The feeding solution for the second reactor contained 87 g L^−1^ glycerol, the supply rates were applied with 29.2 mL h^−1^, 58.4 mL h^−1^ and 87.6 mL h^−1^. In the feeding solution for the third reactor, pyruvate (87 g L^−1^) was used as a carbon source, and it was supplied at rates of 29.7 mL h^−1^, 59.3 mL h^−1^, and 89.0 mL h^−1^. The fourth reactor was provided with a feed solution of 78 g L^−1^ succinate, and the feed profile was set to 32.2 mL h^−1^, 64.4 mL h^−1^, and 69.6 mL h^−1^.

### Rapid sampling

Rapid sampling was performed in order to capture extra- and intracellular metabolite concentrations during metabolic analysis from the analysis reactors and the reference process reactor. Samples for the quantification of extracellular metabolites were taken simultaneously from all four reactors at 1 min, 9 min, 15 min, and 21 min after inoculation via a special vacuum sampling device [58]. The sampled cell suspension was then transferred to precooled centrifuge tubes filled with glass beads for fast cooling. Sampling for the analysis of intracellular metabolite concentrations was conducted at the end of each substrate supply stage (8 min, 14 min, 20 min), using sampling tubes equipped with inner tubes (adapted from [58]) and were filled with 22.5 mL of a quenching fluid (60% methanol, 30 mM triethanolamine (TEA)). One sample was taken from the process bioreactor 10 min after inoculation of the analysis reactors. Prior to sampling, these sampling devices were precooled to −60 °C in a cryostat. The vacuum level of the sampling tubes was reduced to 0.85 bar immediately before the sample was taken. Through small cavities of the inner tubes, the sample was rapidly distributed in the quenching fluid, thus ensuring fast inactivation of the metabolism of the cells. The mixture was then transferred into precooled (−40 °C ethanol/ice-mixture) centrifuge tubes for short-term storage.

### Analytical methods

For the determination of cell dry weight concentrations, 2 mL reaction tubes were dried (3 days, 80 °C) and their empty weight was measured. In triplicates, 2 mL of the sampled cell suspension was pipetted into the pre-weighed tubes, centrifuged (18,940 g, 4 °C, 20 min) and the supernatant was discarded. Left open, the filled reaction tube was dried and weighed again.

High-performance liquid chromatography (HPLC) was used for the quantification of extracellular metabolites. In preparation for the analysis, the samples were cooled on ice for exactly 10 min and centrifuged (18,940 g, 4 °C, 10 min), the supernatant was then filtered through a pore size of 0.2 µM. For the quantification of pelleted l-tryptophan, 1 mL of cooled sample was centrifuged (18,940 g, 4 °C, 10 min), the supernatant was discarded, and the pellet was resuspended 1:2 in a PBS buffer. When fully resuspended, the sample was centrifuged (18,940 g, 4 °C, 10 min), and filtered through a pore size of 0.2 µM. The processed samples were analyzed separately.

Amino acid quantification was performed with an HPLC (Smartline HPLC, Knauer, Berlin, Germany) equipped with a fluorescence detector (RF20A detector, Shimadzu, Kyoto, Japan). Analytes were derivatized by the autosampler (cooled to 8 °C) with o-phtaldialdehyde (OPA), mercaptopropionic acid (MCP), and iodoacetic acid (IAA), all in a 40 mM bicine buffer (pH 10.2) before separation. As a first step, 10 µL of the sample was transferred to a separate destination vial, which was prefilled with 658 µL 0.3 mM MCP. Thereupon, 20 µL of IAA (3.5 mM) was added and mixed. Derivatization was finished by the addition of 70 µL OPA (11 mM in bicine/methanol/MCP (928:7.1:0.1) (v/v/v)) and subsequent mixing. For chromatographic separation 20 µL of the derivatized sample solution were injected into a Gemini column (C_18_ 150 × 4.6 mm, 5 µm; Phenomenex, Torrance, CA, USA). The column temperature was kept at 40 °C, and the flow rate was set to 1 mL min^−1^. During the separation time of 43 min, a gradient was applied using solvent A (20 mM NaH_2_PO_4_, pH 7.6, filtered (0.2 µm)) and solvent B (methanol/acetonitrile/water (45/45/10) (v/v/v)). The following gradient profile was applied: 0–3 min 100% solvent A, 8.5 min 75% solvent A, 30 min 60% solvent A, 30.02 min 0% solvent A, 32 min 0% solvent A, 32.02 20% solvent A, 34 min 20% solvent A, 38 min 100% solvent A, 43 min 100% solvent A.

Organic acids and alcohols were quantified by an HPLC system (LC-2030C Plus, Shimadzu, Kyoto, Japan) equipped with a refractive index detector (RID 20A, Shimadzu, Kyoto, Japan) at 950 nm. The analytes were separated on an Aminex HPLX-87H column (BioRad, Munich, Germany) at 65 °C at an isocratic flow of 0.6 mL min^−1^ (5 mM H_2_SO_4_). A volume of 20 µL was injected on the column.

Ammonia was quantified with an enzymatic assay from Boehringer Mannheim/R-Biopharm (Darmstadt, Germany; Kits No 10716251035 and 11112732035). Methylglyoxal was also quantified enzymatically with an assay purchased from Biovision (Milpitas, CA, USA; Methylglyoxal Assay Kit; Kits No K500). A deproteinizing sample preparation kit (Milpitas, CA, USA; Deproteinizing Sample Preparation Kit II; Kits No K823) was used for sample preparation. For accurate readings, a background curve was measured, covering the entire concentration range.

### Metabolome quantification

The samples used for the quantification of intracellular metabolites were cooled (−40 °C) until further progressed. First, the diluted cell suspension was mixed thoroughly, and 1 mL was extracted at 95 °C in 2 mL of TEA buffer (30 mM, pH 7). Extraction was performed in quadruplets; two mixtures were supplemented with 350 µL of U-^13^C cell extract as internal standard (undiluted and diluted 1:10, respectively), and in the two remaining mixtures nothing was added. The U-^13^C cell extract was prepared according to Weiner [59], with *E. coli* NT1446. After 5 min of extraction, the samples were cooled on ice and centrifuged (20 min, 3,260 g). The supernatant was separated and stored at −80 °C. A UHPLC-MS/MS method adapted from Buescher [60] was applied to analyze the metabolome. A mass spectrometry system (TSQ Vantage, Thermo Fisher Scientific, Waltham; MA, USA) was used as a detector. The analytes were separated chromatographically on an Acquidity HSS T3 column (150 mm × 2.1 mm × 1.8 µm; Waters Corporation, Milford, USA) at 40 °C for 36 min, applying a gradient of solvent A (10 mM tributylamine, 15 mM acetic acid, 5% (v/v) methanol) and solvent B (100% isopropanol). The sample injection volume was 20 µL. The solvents used were of ultrapure MS quality. The ionization of analytes was achieved at a spray voltage of 2.0 kV and a vaporizer temperature of 400 °C. The sheath gas pressure was set to 5.0, the aux gas pressure to 20.0 (arbitrary units), and the capillary was heated to 380 °C.

### Thermodynamics-based flux analysis

A python implemented package, published by Salvy [15], was used to perform thermodynamics-based flux analysis. The genome-scale model used was *i*JO1366 [61]. Extracellular rates (substrate uptake, production, by-product formation, and respiration were used to constrain the solution space, and maximization of growth was chosen as the objective function. Prior to TFA, a loopless Flux Variability Analysis was performed, and null values were used to further constrain the thermodynamic estimations. Thermodynamic parameters were adapted via in vivo conditions, so the extracellular pH was set to 7.0 and the ionic strength to 0.15 M. Thermodynamic information was either already included in the model or taken from the Equilibrator database [62]. The measured metabolome data was also included. The variance criterium for the optimal solution was chosen to be 99.9%. Besides TFA solution, ranges for each reaction in the model as well as feasible ranges for metabolite concentrations and Gibbs reaction energies were obtained. The solution space was investigated statistically by means of sampling with the OptGpSampler [63]. Metabolic control analysis (MCA) was calculated according to the linearization approach proposed by Visser and Heijnen [48]. The production process on a 15 L scale in the time frame of metabolic analysis served as a reference point for the linearization of mass balance equations. All of the 12 metabolic equilibria achieved during the metabolic analysis were included in the calculations. A reduced metabolic model was used with 53 reactions and 59 metabolites from glycolysis, glycerol metabolism, MGO pathway, TCA cycle, PPP, l-serine biosynthesis, and biosynthesis of aromatic amino acids. Further details about the mathematical background can be obtained from the follow publications: [4, 42, 48]. The resulting FCCs represent the effects of a 1% increase in the enzymatic activity on a certain flux in the metabolic network. Activating effects are described by positive values and inhibitory effects by negative signs. Monte Carlo sampling (10,000 cycles) was performed in order to take uncertainties of metabolite concentrations, metabolic fluxes, and Gibbs reaction energies into account [64]. MATLAB 2021b (Mathworks, Natick, MA, USA) was used.

### RT-qPCR analysis (Stuttgart)

The qRT-PCR was done as previously described [44]. The primers used for this analysis are listed in Table 1. The *ftsZ* gene was used as an endogenous calibrator for the analysis. The data were analyzed using the qPCRsoft 3.4 software application (Analytik Jena).

## Supplementary Information

**Additional file 1:** **Figure S1.** Schematic representation of central carbon metabolism and L-tryptophan biosynthesis pathway. Depicted are the metabolites glucose-6-phosphate (G6P), fructose 1,6-bisphosphate (FBP), glyceraldehyde 3-phosphate (GAP), 3-phospho-D-glycerate (3PG), phosphoenolpyruvate (PEP), pyruvate (PYR), 3-phosphohydroxypyruvate (3PHP), O-phospho-L-serine (L-PSer) and L-serine (L-ser) from glycolysis and glycerol metabolism (GLYC & GLYK), 6-phosphoglucono-1,5-lactone (6PG), ribulose 5-phosphate (Ru5P), xylulose 5-phosphate (X5P), ribose 5-phosphate (R5P), sedoheptulose 7-phosphate (S7P), erythrose 4-phosphate (E4P), fructose 6-phosphate (F6P) and 5-phospho-alpha-D-ribose 1-diphosphate (PRPP) from the pentose-phosphate-pathway (PPP), Acetyl-Coa (AcCoA) from the citric acid cycle (TCA), chorismate (CHOR), 3-dehydroquinate (3DHQ), 3-dehydroshikimate (3DHS), shikimate (SHIK) and chorismate (CHOR) from the chorismate biosynthesis pathway (CHOR) as well as anthranilate (ANTH), N-(5-phospho-D-ribosyl)anthranilate (PRAN), 1-(2-Carboxyphenylamino)-1-deoxy-D-ribulose 5-phosphate (CDRP), 3-Indolyl-glycerol 3-phosphate (IGP), indole (IND) and L-tryptophan (L-trp) from the L-tryptophan biosynthesis pathway (L-trp). Relevant genes are denoted in a white font with a coloured background. Genes, which were introduced into the new L-trpytophan producer strains (based on the initial producer strain *E. coli* NT1259) in additional genomic copies are highlighted in colors: *serB* in orange, *aroB* in green, *trpC*, coding for a monofunctional version of indole-glycerolphosphate synthase in yellow and *trpB* as well as *trpA* in blue. The modifications resulted in the new producer strains NT1259 *trpC*_*mt*_(NT1405), NT1259 *trpBA* (NT1438), NT1259 *trpBA trpC*_mt_ (NT1439), NT1259 *trpBA trpC*_*mt*_ *aroB* (NT1445), NT1259 *trpBA trpC*_mt_ *serB* (NT1444), NT1259 *trpBA* *trpC*_mt_ *aroB serB* (NT1446), which were used alongside the reference strain NT1259 in this study. **Figure S2.** Total amounts of L-tryptophan (L-trp) and cell dry weight (CDW) during fed-batch production of L-tryptophan with *E. coli* NT1259 pF112*aroFBL*_Kan_ on a 15 L scale (37 °C, pH 7.0, DO > 30% air saturation) of three different processes (run 1-3). Vertical solid black lines indicate (i) the end of the batch phase (~10.4 h) and (ii) the beginning of the constant feeding phase/addition of IPTG (~45.1 h). The broken red line marks the process time for cell sampling (~48.7 h). **Figure S3.** Total amounts of L-tryptophan (L-trp) and cell dry weight (CDW) during fed-batch production of L-tryptophan with *E. coli* NT1405 pF112*aroFBL*_Kan_ on a 15 L scale (37 °C, pH 7.0, DO > 30% air saturation). Vertical solid black lines indicate (i) the end of the batch phase (9.9 h) and (ii) the beginning of the constant feeding phase/addition of IPTG (45.1 h). The broken red line marks the process time for cell sampling (49.5 h). **Figure S4.** Total amounts of L-tryptophan (L-trp) and cell dry weight (CDW) during fed-batch production of L-tryptophan with *E. coli* NT1438 pF112*aroFBL*_Kan_ on a 15 L scale (37 °C, pH 7.0, DO > 30% air saturation). Vertical solid black lines indicate (i) the end of the batch phase (10.4 h) and (ii) the beginning of the constant feeding phase/addition of IPTG (45.1 h). The broken red line marks the process time for cell sampling (48.8 h). **Figure S5.** Total amounts of L-tryptophan (L-trp) and cell dry weight (CDW) during fed-batch production of L-tryptophan with *E. coli* NT1439 pF112*aroFBL*_Kan_ on a 15 L scale (37 °C, pH 7.0, DO > 30% air saturation). Vertical solid black lines indicate (i) the end of the batch phase (9.0 h) and (ii) the beginning of the constant feeding phase/addition of IPTG (44.7 h). The broken red line marks the process time for cell sampling (48.2 h). **Figure S6. **Total amounts of L-tryptophan (L-trp) and cell dry weight (CDW) during fed-batch production of L-tryptophan with *E. coli* NT1445 pF112*aroFBL*_Kan_ on a 15 L scale (37 °C, pH 7.0, DO > 30% air saturation). Vertical solid black lines indicate (i) the end of the batch phase (10.6 h) and (ii) the beginning of the constant feeding phase/addition of IPTG (45.7 h). The broken red line marks the process time for cell sampling (50.0 h). **Figure S7.** Total amounts of L-tryptophan (L-trp) and cell dry weight (CDW) during fed-batch production of L-tryptophan with *E. coli* NT1444 pF112*aroFBL*_Kan_ on a 15 L scale (37 °C, pH 7.0, DO > 30% air saturation). Vertical solid black lines indicate (i) the end of the batch phase (18.8 h) and (ii) the beginning of the constant feeding phase/addition of IPTG (49.7 h). The broken red line marks the process time for cell sampling (52.8 h). **Figure S8.** Total amounts of L-tryptophan (L-trp) and cell dry weight (CDW) during fed-batch production of L-tryptophan with *E. coli* NT1446 pF112*aroFBL*_Kan_ on a 15 L scale (37 °C, pH 7.0, DO > 30% air saturation) of three different processes (run 1-3). Vertical solid black lines indicate (i) the end of the batch phase (~10.1 h) and (ii) the beginning of the constant feeding phase/addition of IPTG (~45.6 h). The broken red line marks the process time for cell sampling (~48.3 h). **Figure S9. **Fed-batch production of L-tryptophan with *E. coli* NT1446 pF112*aroFBL*_Kan_ on a 15 L scale (37 °C, pH 7.0, DO > 30% air saturation). A: Concentrations (unit: g L^−1^) of glycerol and ammonium. B: Concentrations (unit: g L^−1^) of L-tyrosine, L-phenylalanine, D-lactate, malate and pyruvate. Vertical solid black lines indicate (i) the end of the batch phase (9.83 h) and (ii) the beginning of the constant feeding phase/addition of IPTG (43.9 h). The broken red line marks the process time for cell sampling for the parallel metabolic perturbation studies (47.9 h). **Figure S10. **Heat map illustrating flux distributions (unit: mmol g_CDW_^−1^ h^−1^) in glycolysis and glycerol metabolism (GLYC & GLYK), methylglyoxal pathway (MGO), TCA cycle (TCA), pentose-phosphate-pathway (PPP), L-serine biosynthesis (L-ser), chorismate biosynthesis (CHOR), L-phenylalanine and L-tyrosine biosynthesis (L-phe/ L-tyr) and L-tryptophan production (L-trp) derived by thermodynamics-based flux analysis (pyTFA), restricted by measured extracellular rates during the parallel short-term perturbation experiments in stirred-tank bioreactors for metabolic analysis of L-tryptophan producing *E. coli* cells. Fluxes are depicted for the reference L-tryptophan production process with glycerol as sole carbon source (Ref) and the analysis reactors with glycerol (Glyc), glucose (Gluc), pyruvate (Pyr) as well as succinate (Suc). Flux directions are defined as in the model *i*JO1366. **Figure S11.** Estimated Gibbs‘ energies of reactions (unit: kJ mol-1) for reactions of glycolysis and glycerol metabolism (GLYC & GLYK), methylglyoxal pathway (MGO), TCA cycle (TCA), pentose-phosphate-pathway (PPP), L-serine biosynthesis (L-ser), chorismate biosynthesis (CHOR), L-phenylalanine and L-tyrosine biosynthesis (L-phe/ L-tyr) and L-tryptophan production (L-trp) of cells in the L-tryptophan fed-batch production process at the process time chosen for metabolic analysis, derived by thermodynamic flux analysis (pyTFA) in consideration of measured intracellular metabolite concentrations and Gibbs’ free energies of formation for intracellular pH of pH 7.5 and ionic strength of 0.15 M. Reaction energies are depicted for positive flux directions, estimated with thermodynamics-based flux analysis for the reference state. **Figure S12.** RT-qPCR analyzed relative gene expression (without unit) of *mgsA* gene relative to *ftsZ* gene of samples from the four analysis reactors with the supplied carbon sources glycerol (MA Glyc), glucose (MA Gluc), pyruvate (MA Pyr) and succinate (MA Suc). **Figure S13.** RT-qPCR analyzed relative gene expression (without unit) of the genes *gloA, gloB, gloC, yeiG* and *dld* relative to *ftsZ* gene of samples from the four analysis reactors with the supplied carbon sources glycerol (MA Glyc), glucose (MA Gluc), pyruvate (MA Pyr) and succinate (MA Suc). **Figure S14.** RT-qPCR analyzed relative gene expression (without unit) of the genes *yaijL* and *yhbO* relative to *ftsZ* gene of samples from the four analysis reactors with the supplied carbon sources glycerol (MA Glyc), glucose (MA Gluc), pyruvate (MA Pyr) and succinate (MA Suc).

## Data Availability

The datasets supporting the conclusions of this article are included within the article and its additional file.

## Abbreviations

3DHS: 3-Dehydroshikimate
ANTH: Anthranilate
anprt: Anthranilate phosphoribosyltransferase
ans: Anthranilate synthase
CDRP: 1-(2-Carboxyphenylamino)-1-deoxy-D-ribulose 5-phosphate (CDRP)
CDW: Cell dry weight
CHOR: Chorismate
chors: Chorismate synthase
CPR: Carbon dioxide production rates (CPR)
DHAP: Dihydroxyacetone-phosphate
dhqs: 3-Dehydroquinate synthase
E. coli: Escherichia coli
eno: Enolase
FBP: Fructose 1,6-bisphosphate
FCC: Flux control coefficient
gly3pdh: Glycerol-3-phosphate dehydrogenase
glyk: Glycerol kinase
glyox: Glyoxylase
HPLC: High-performance liquid chromatography
IAA: Iodoacetic acid
igps: Indol-glycerolphosphate synthase
IPTG: Isopropyl-β-D-thiogalactopyranosid
ldh: Lactate dehydrogenase
MCA: Metabolic control analysis
MCP: Mercaptopropionic acid
MGO: Methylglyoxal
mgsa: Methylglyoxal synthase
*M. tuber*: *Mycobacterium tuberculosis*
OPA: o-phtaldialdehyde
OUR: Oxygen uptake
pgm: Phosphoglycerate mutase
ppndh: Prephenate dehydratase
PPP: Pentose phosphate pathway
prali: Phosphoribosylanthranilate isomerase
prpps: Phosphoribosylpyrophosphate synthetase
pscvt: 3-Phosphoshikimate 1-carboxyvinyltransferase
pser_L: Phospho l-serine
psp_L: Phosphoserine phosphatase
RMT: Rapid media transition
S3P: Shikimate 3-phosphate
TCA: Citric acid
TEA: Triethanolamine
TFA: Thermodynamics-based flux analysis
trps2: Tryptophan synthase
