# Supplementary material for: Metabolic control analysis enables rational improvement of E. coli l-tryptophan producers but methylglyoxal formation limits glycerol-based production
Source: Microb Cell Fact. 2022 Oct 4;21:201. doi: 10.1186/s12934-022-01930-1 (PMC9531422; doi:10.1186/s12934-022-01930-1)
Supplement: Supplementary file 1 — Additional file 1: Figure S1. Schematic representation of central carbon metabolism and L-tryptophan biosynthesis pathway. Depicted are the metabolites glucose-6-phosphate (G6P), fructose 1,6-bisphosphate (FBP), glyceraldehyde 3-phosphate (GAP), 3-phospho-D-glycerate (3PG), phosphoenolpyruvate (PEP), pyruvate (PYR), 3-phosphohydroxypyruvate (3PHP), O-phospho-L-serine (L-PSer) and L-serine (L-ser) from glycolysis and glycerol metabolism (GLYC & GLYK), 6-phosphoglucono-1,5-lactone (6PG), ribulose 5-phosphate (Ru5P), xylulose 5-phosphate (X5P), ribose 5-phosphate (R5P), sedoheptulose 7-phosphate (S7P), erythrose 4-phosphate (E4P), fructose 6-phosphate (F6P) and 5-phospho-alpha-D-ribose 1-diphosphate (PRPP) from the pentose-phosphate-pathway (PPP), Acetyl-Coa (AcCoA) from the citric acid cycle (TCA), chorismate (CHOR), 3-dehydroquinate (3DHQ), 3-dehydroshikimate (3DHS), shikimate (SHIK) and chorismate (CHOR) from the chorismate biosynthesis pathway (CHOR) as well as anthranilate (ANTH), N-(5-phospho-D-ribosyl)anthranilate (PRAN), 1-(2-Carboxyphenylamino)-1-deoxy-D-ribulose 5-phosphate (CDRP), 3-Indolyl-glycerol 3-phosphate (IGP), indole (IND) and L-tryptophan (L-trp) from the L-tryptophan biosynthesis pathway (L-trp). Relevant genes are denoted in a white font with a coloured background. Genes, which were introduced into the new L-trpytophan producer strains (based on the initial producer strain E. coli NT1259) in additional genomic copies are highlighted in colors: serB in orange, aroB in green, trpC, coding for a monofunctional version of indole-glycerolphosphate synthase in yellow and trpB as well as trpA in blue. The modifications resulted in the new producer strains NT1259 trpCmt(NT1405), NT1259 trpBA (NT1438), NT1259 trpBA trpCmt (NT1439), NT1259 trpBA trpCmt aroB (NT1445), NT1259 trpBA trpCmt serB (NT1444), NT1259 trpBA trpCmt aroB serB (NT1446), which were used alongside the reference strain NT1259 in this study. Figure S2. Total amounts of L-tryptophan (L-trp) [file 12934_2022_1930_MOESM1_ESM.pdf]

Microbial Cell Factories

Metabolic control analysis enables rational improvement of *E. coli* L-tryptophan producers but methylglyoxal formation limits glycerol-based production

Kristin Schoppel[1], Natalia Trachtmann[2], Emil Korzin[1], Angelina Tzanavari[1], Georg A. Sprenger[2], Dirk Weuster-Botz[1]

[1] Technical University of Munich, Institute of Biochemical Engineering, Boltzmannstraße 15, 85748 Garching, Germany

[2] University of Stuttgart, Institute of Microbiology, Allmandring 31, 70569, Stuttgart, Germany

Email: dirk.weuster-botz@tum.de

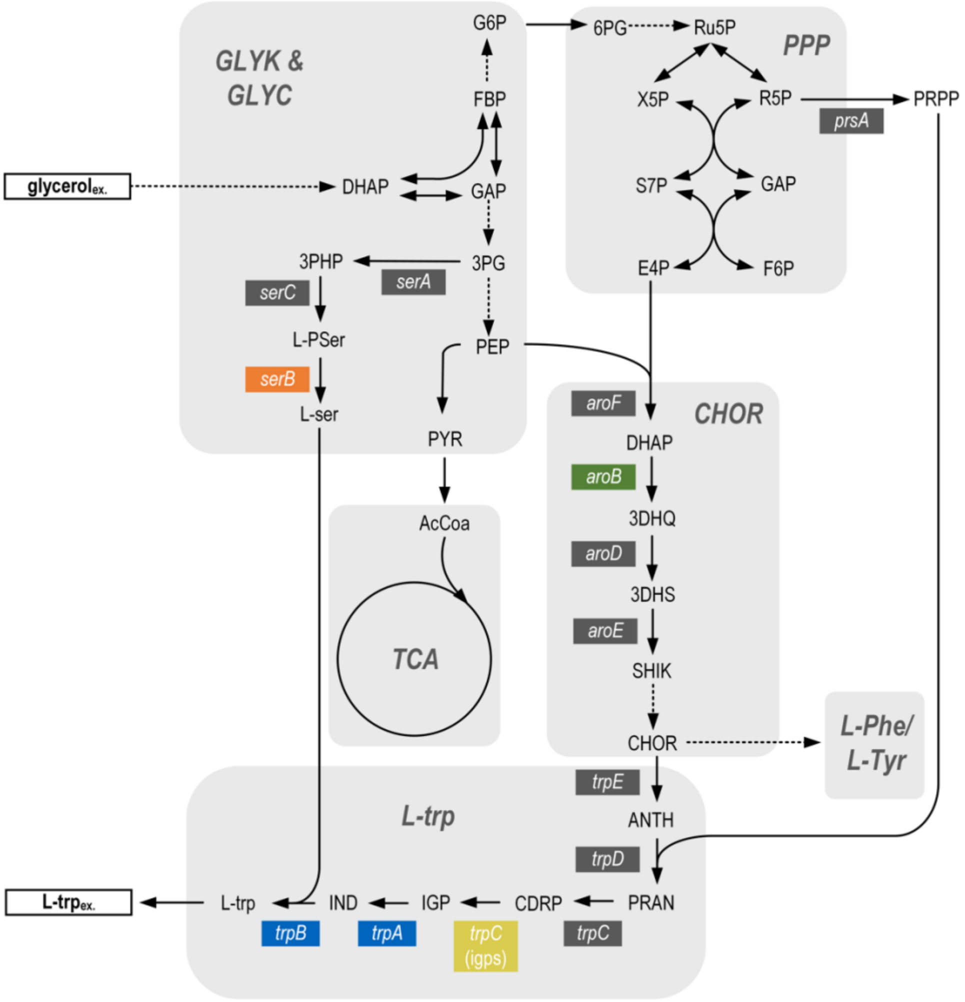

Figure 1: Schematic representation of central carbon metabolism and L-tryptophan biosynthesis pathway. Depicted are the metabolites glucose-6-phosphate (G6P), fructose 1,6-bisphosphate (FBP), glyceraldehyde 3-phosphate (GAP), 3-phospho-D-glycerate (3PG), phosphoenolpyruvate (PEP), pyruvate (PYR), 3-phosphohydroxypyruvate (3PHP), O-phospho-L-serine (L-PSer) and L-serine (L-ser) from glycolysis and glycerol metabolism (*GLYC & GLYK*), 6-phosphoglucono-1,5-lactone (6PG), ribulose 5-phosphate (Ru5P), xylulose 5-phosphate (X5P), ribose 5-phosphate (R5P), sedoheptulose 7-phosphate (S7P), erythrose 4-phosphate (E4P), fructose 6-phosphate (F6P) and 5-phospho-alpha-D-ribose 1-diphosphate (PRPP) from the pentose-phosphate-pathway (*PPP*), Acetyl-Coa (AcCoA) from the citric acid cycle (*TCA*), chorismate (CHOR), 3-dehydroquinate (3DHQ), 3-dehydroshikimate (3DHS), shikimate (SHIK) and chorismate (CHOR) from the chorismate biosynthesis pathway (*CHOR*) as well as anthranilate (ANTH),

N-(5-phospho-D-ribosyl)anthranilate (PRAN), 1-(2-Carboxyphenylamino)-1-deoxy-D-ribulose 5-phosphate (CDRP), 3-Indolyl-glycerol 3-phosphate (IGP), indole (IND) and L-tryptophan (L-trp) from the L-tryptophan biosynthesis pathway (*L-trp*). Relevant genes are denoted in a white font with a coloured background. Genes, which were introduced into the new L-trpytophan producer strains (based on the initial producer strain *E. coli* NT1259) in additional genomic copies are highlighted in colors: *serB* in orange, *aroB* in green, *trpC*, coding for a monofunctional version of indole-glycerolphosphate synthase in yellow and *trpB* as well as *trpA* in blue. The modifications resulted in the new producer strains NT1259 *trpC$_{mt}$* (NT1405), NT1259 *trpBA* (NT1438), NT1259 *trpBA trpC$_{mt}$* (NT1439), NT1259 *trpBA trpC$_{mt}$ aroB* (NT1445), NT1259 *trpBA trpC$_{mt}$ serB* (NT1444), NT1259 *trpBA trpC$_{mt}$ aroB serB* (NT1446), which were used alongside the reference strain NT1259 in this study.

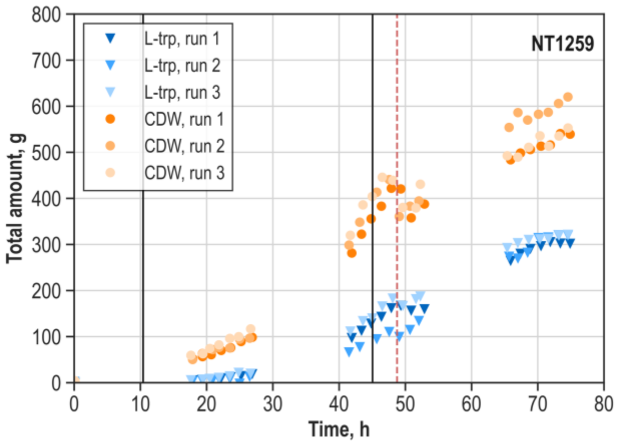

Fig. 1 Total amounts of L-tryptophan (L-trp) and cell dry weight (CDW) during fed-batch production of L-tryptophan with *E. coli* NT1259 pF112*aroFBL*$_{Kan}$ on a 15 L scale (37 °C, pH 7.0, DO > 30% air saturation) of three different processes (run 1-3). Vertical solid black lines indicate (i) the end of the batch phase (~10.4 h) and (ii) the beginning of the constant feeding phase/addition of IPTG (~45.1 h). The broken red line marks the process time for cell sampling (~48.7 h).

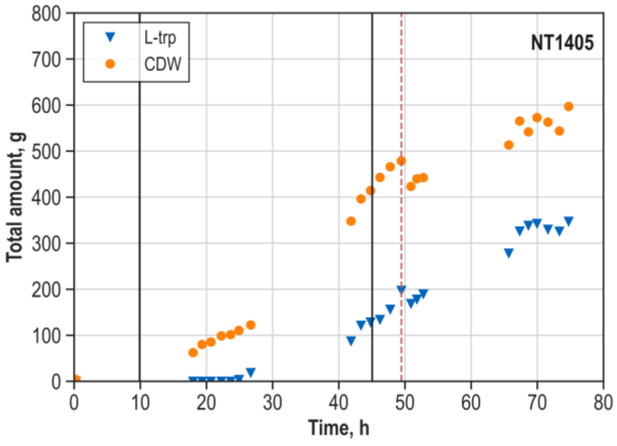

Fig. 2 Total amounts of L-tryptophan (L-trp) and cell dry weight (CDW) during fed-batch production of L-tryptophan with *E. coli* NT1405 pF112*aroFBL*$_{Kan}$ on a 15 L scale (37 °C, pH 7.0, DO > 30% air saturation). Vertical solid black lines indicate (i) the end of the batch phase (9.9 h) and (ii) the beginning of the constant feeding phase/addition of IPTG (45.1 h). The broken red line marks the process time for cell sampling (49.5 h).

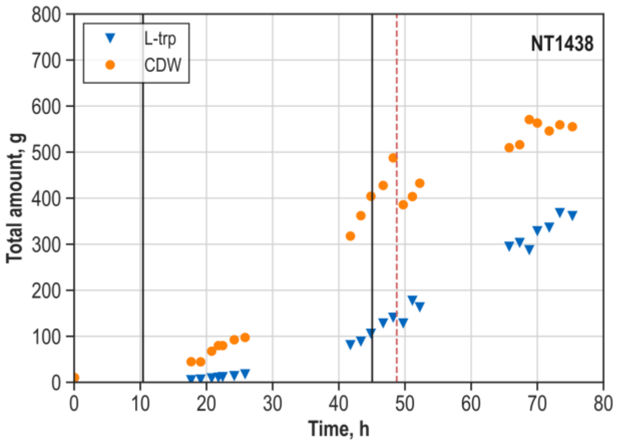

Fig. 3 Total amounts of L-tryptophan (L-trp) and cell dry weight (CDW) during fed-batch production of L-tryptophan with *E. coli* NT1438 pF112*aroFBL*$_{\text{Kan}}$ on a 15 L scale (37 °C, pH 7.0, DO > 30% air saturation). Vertical solid black lines indicate (i) the end of the batch phase (10.4 h) and (ii) the beginning of the constant feeding phase/addition of IPTG (45.1 h). The broken red line marks the process time for cell sampling (48.8 h).

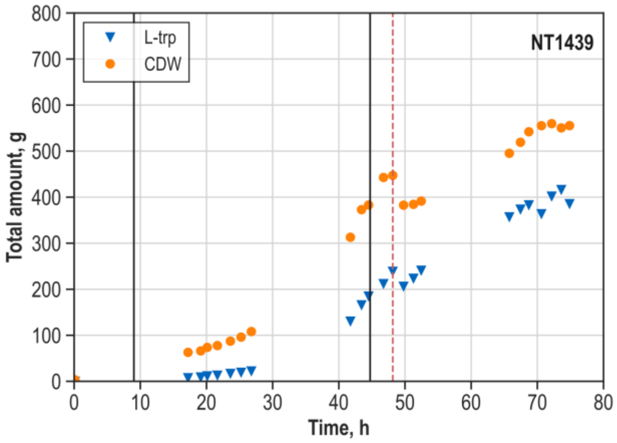

Fig. 4 Total amounts of L-tryptophan (L-trp) and cell dry weight (CDW) during fed-batch production of L-tryptophan with *E. coli* NT1439 pF112*aroFBL*$_{\text{Kan}}$ on a 15 L scale (37 °C, pH 7.0, DO > 30% air saturation). Vertical solid black lines indicate (i) the end of the batch phase (9.0 h) and (ii) the beginning of the constant feeding phase/addition of IPTG (44.7 h). The broken red line marks the process time for cell sampling (48.2 h).

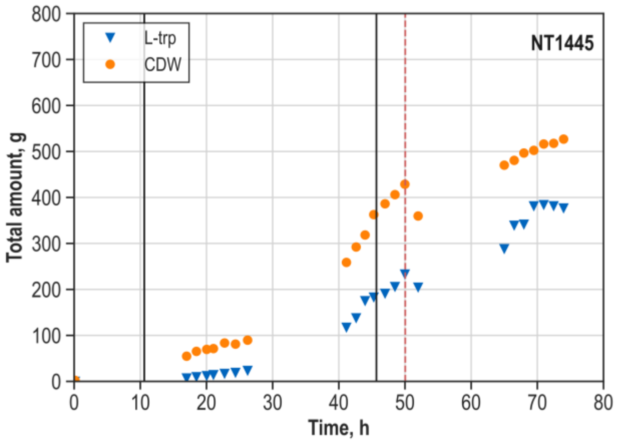

Fig. 5 Total amounts of L-tryptophan (L-trp) and cell dry weight (CDW) during fed-batch production of L-tryptophan with *E. coli* NT1445 pF112*aroFBL*$_{\text{Kan}}$ on a 15 L scale (37 °C, pH 7.0, DO > 30% air saturation). Vertical solid black lines indicate (i) the end of the batch phase (10.6 h) and (ii) the beginning of the constant feeding phase/addition of IPTG (45.7 h). The broken red line marks the process time for cell sampling (50.0 h).

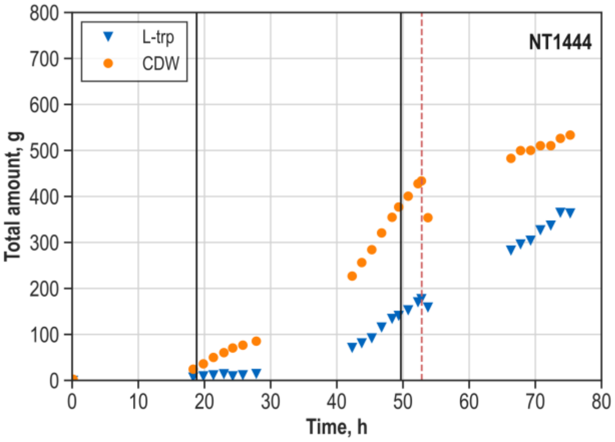

Fig. 6 Total amounts of L-tryptophan (L-trp) and cell dry weight (CDW) during fed-batch production of L-tryptophan with *E. coli* NT1444 pF112*aroFBL*$_{Kan}$ on a 15 L scale (37 °C, pH 7.0, DO > 30% air saturation). Vertical solid black lines indicate (i) the end of the batch phase (18.8 h) and (ii) the beginning of the constant feeding phase/addition of IPTG (49.7 h). The broken red line marks the process time for cell sampling (52.8 h).

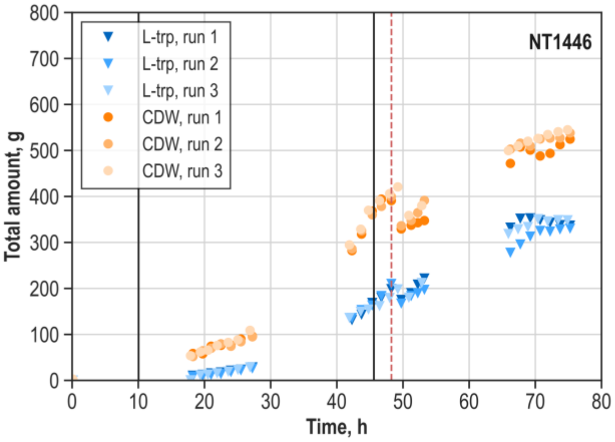

Fig. 7 Total amounts of L-tryptophan (L-trp) and cell dry weight (CDW) during fed-batch production of L-tryptophan with *E. coli* NT1446 pF112*aroFBL*$_{Kan}$ on a 15 L scale (37 °C, pH 7.0, DO > 30% air saturation) of three different processes (run 1-3). Vertical solid black lines indicate (i) the end of the batch phase (~10.1 h) and (ii) the beginning of the constant feeding phase/addition of IPTG (~45.6 h). The broken red line marks the process time for cell sampling (~48.3 h).

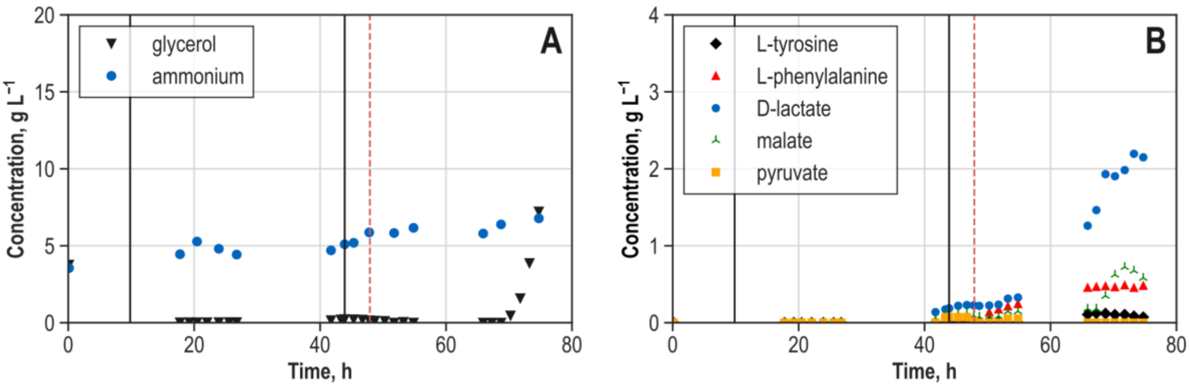

Fig. 8 Fed-batch production of L-tryptophan with *E. coli* NT1446 pF112*aroFBL*$_{\mathrm{Kan}}$ on a 15 L scale (37 °C, pH 7.0, DO > 30% air saturation). A: Concentrations (unit: g L^{-1}) of glycerol and ammonium. B: Concentrations (unit: g L^{-1}) of L-tyrosine, L-phenylalanine, D-lactate, malate and pyruvate. Vertical solid black lines indicate (i) the end of the batch phase (9.83 h) and (ii) the beginning of the constant feeding phase/addition of IPTG (43.9 h). The broken red line marks the process time for cell sampling for the parallel metabolic perturbation studies (47.9 h).

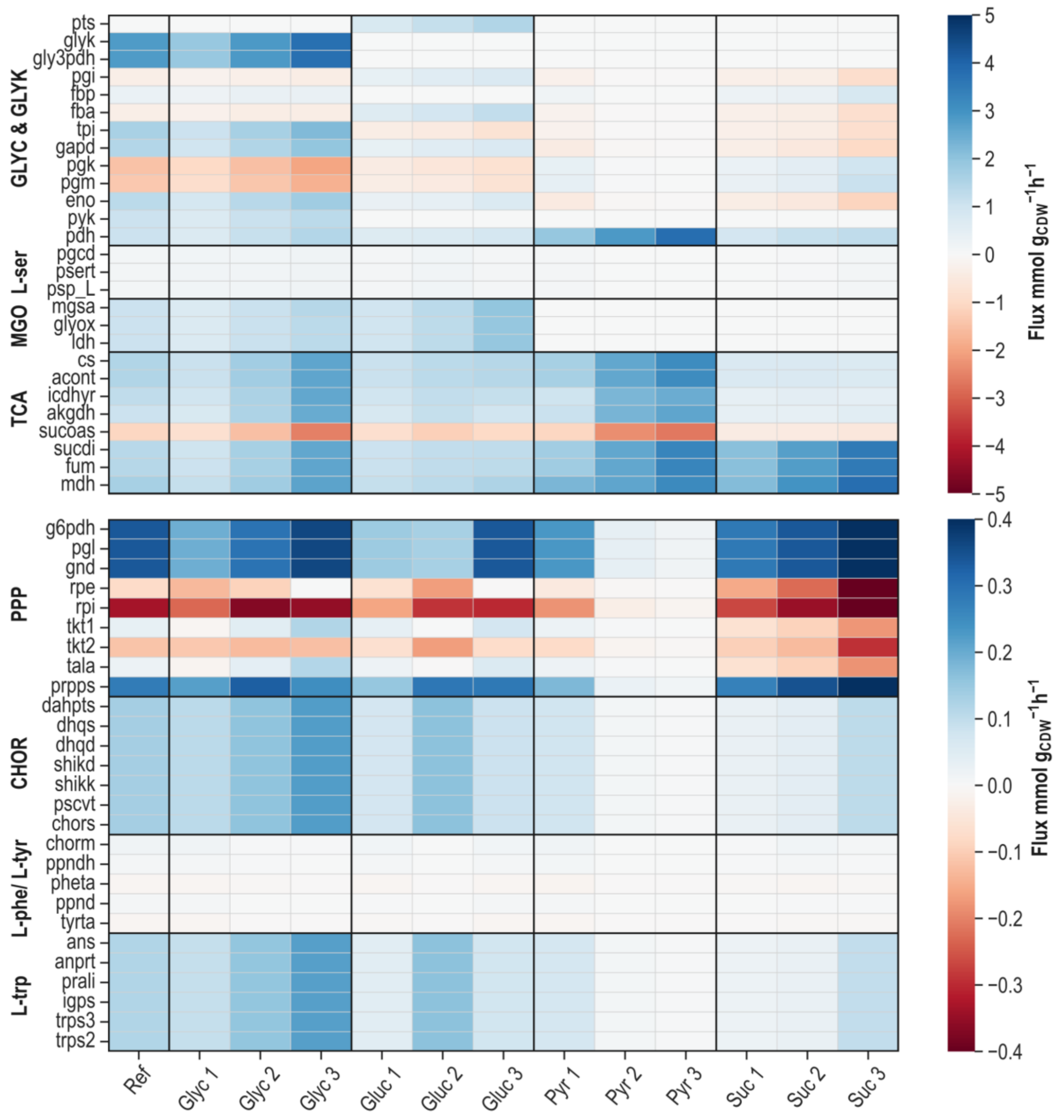

Fig. 9 Heat map illustrating flux distributions (unit: mmol g_{CDW}^{-1} h^{-1}) in glycolysis and glycerol metabolism (GLYC & GLYK), methylglyoxal pathway (MGO), TCA cycle (TCA), pentose-phosphate-pathway (PPP), L-serine biosynthesis (L-ser), chorismate biosynthesis (CHOR), L-phenylalanine and L-tyrosine biosynthesis (L-phe/ L-tyr) and L-tryptophan production (L-trp) derived by thermodynamics-based flux analysis (pyTFA), restricted by measured extracellular rates during the parallel short-term perturbation experiments in stirred-tank bioreactors for metabolic analysis of L-tryptophan producing *E. coli* cells. Fluxes are depicted for the reference L-tryptophan production process with glycerol as sole carbon source (Ref) and the analysis reactors with glycerol (Glyc), glucose (Gluc), pyruvate (Pyr) as well as succinate (Suc). Flux directions are defined as in the model *i*JO1366.

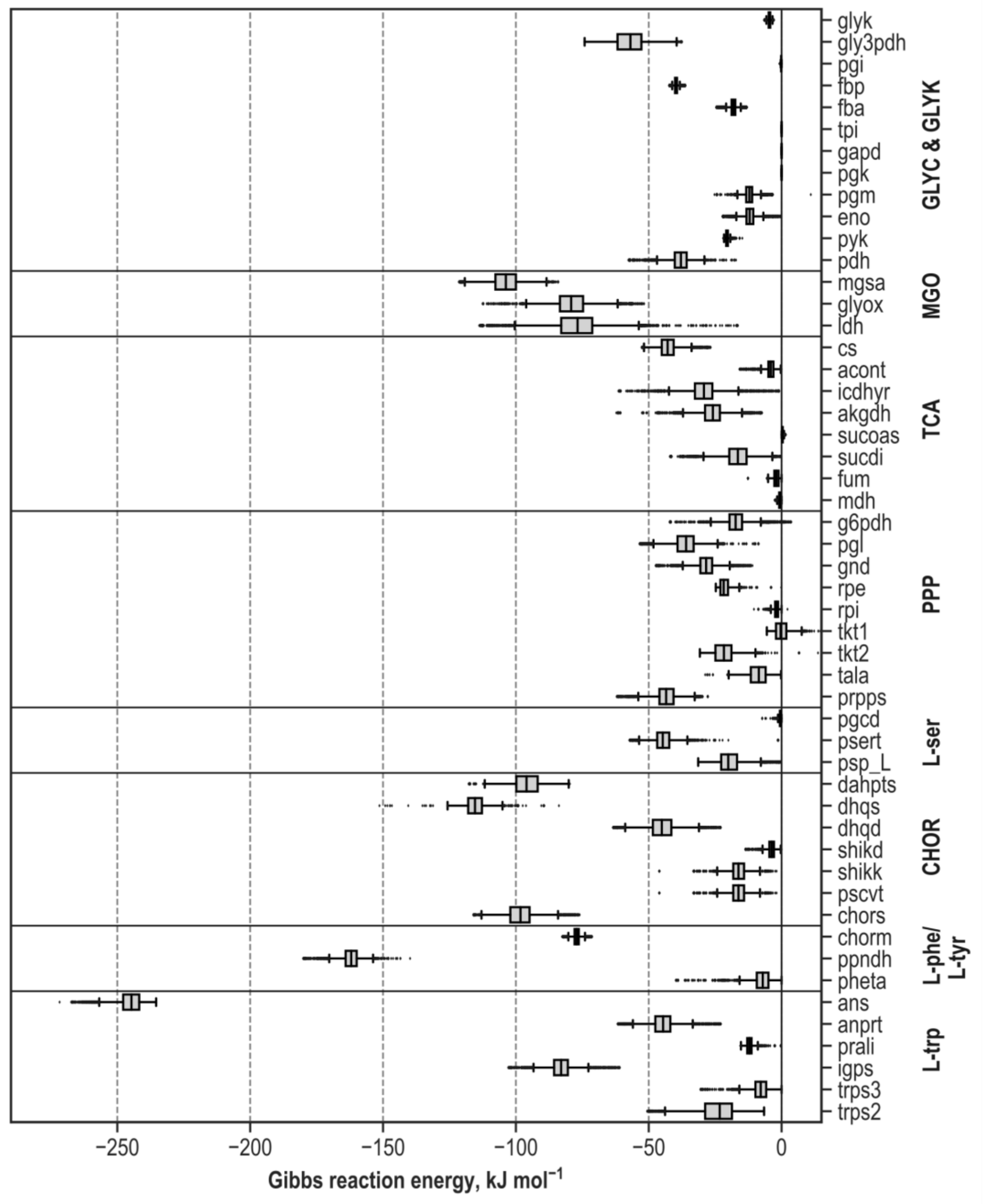

Fig. 10 Estimated Gibbs' energies of reactions (unit: kJ mol-1) for reactions of glycolysis and glycerol metabolism (GLYC & GLYK), methylglyoxal pathway (MGO), TCA cycle (TCA), pentose-phosphate-pathway (PPP), L-serine biosynthesis (L-ser), chorismate biosynthesis (CHOR), L-phenylalanine and L-tyrosine biosynthesis (L-phe/ L-tyr) and L-tryptophan production (L-trp) of cells in the L-tryptophan fed-batch production process at the process time chosen for metabolic analysis, derived by thermodynamic flux analysis (pyTFA) in consideration of measured intracellular metabolite concentrations and Gibbs' free energies of formation for intracellular pH of pH 7.5 and ionic strength of 0.15 M. Reaction energies are depicted for positive flux directions, estimated with thermodynamics-based flux analysis for the reference state.

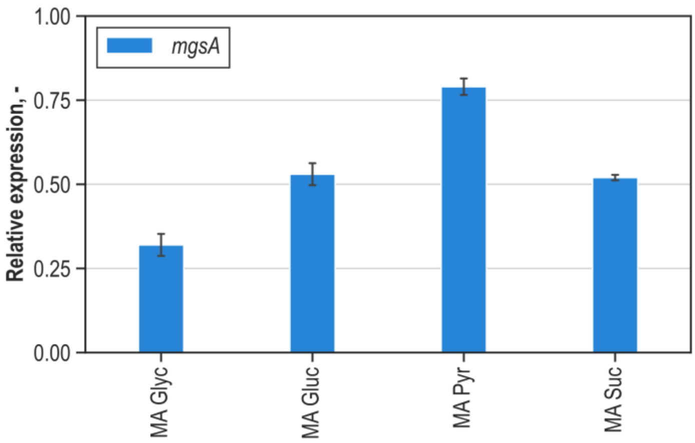

Fig. 11 RT-qPCR analyzed relative gene expression (without unit) of *mgsA* gene relative to *ftsZ* gene of samples from the four analysis reactors with the supplied carbon sources glycerol (MA Glyc), glucose (MA Gluc), pyruvate (MA Pyr) and succinate (MA Suc)**.**

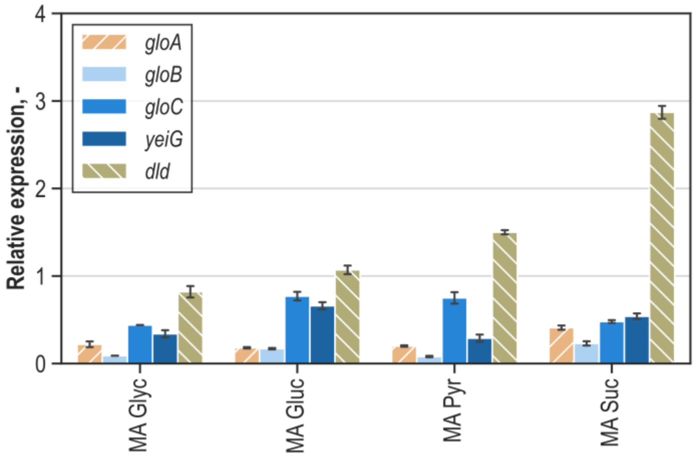

Fig. 12 RT-qPCR analyzed relative gene expression (without unit) of the genes *gloA*, *gloB*, *gloC*, *yeiG* and *dld* relative to *ftsZ* gene of samples from the four analysis reactors with the supplied carbon sources glycerol (MA Glyc), glucose (MA Gluc), pyruvate (MA Pyr) and succinate (MA Suc)**.**

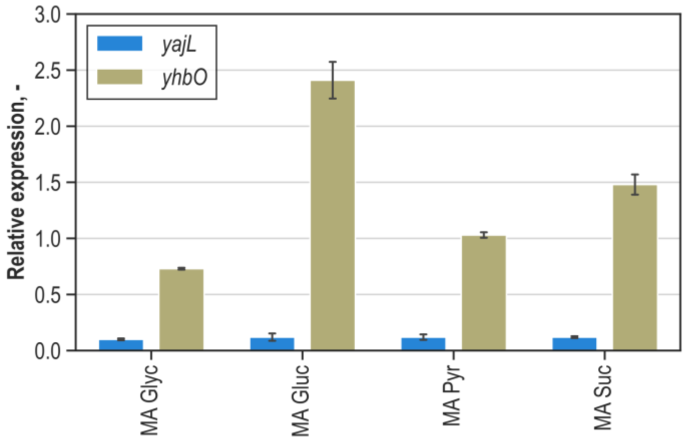

Fig. 13 RT-qPCR analyzed relative gene expression (without unit) of the genes *yaijL* and *yhbO* relative to *ftsZ* gene of samples from the four analysis reactors with the supplied carbon sources glycerol (MA Glyc), glucose (MA Gluc), pyruvate (MA Pyr) and succinate (MA Suc).
